# Stochastic modeling of influenza spread dynamics with recurrences

**DOI:** 10.1371/journal.pone.0231521

**Published:** 2020-04-21

**Authors:** John Whitman, Ciriyam Jayaprakash

**Affiliations:** Department of Physics, The Ohio State University, Columbus, Ohio, United States of America; International Prevention Research Institute, FRANCE

## Abstract

We present results of a study of a simple, stochastic, agent-based model of influenza A infection, simulating its dynamics over the course of one flu season. Building on an early work of Bartlett, we define a model with a limited number of parameters and rates that have clear epidemiological interpretation and can be constrained by data. We demonstrate the occurrence of recurrent behavior in the infected number [more than one peak in a season], which is observed in data, in our simulations for populations consisting of cohorts with strong intra- and weak inter-cohort transmissibility. We examine the dependence of the results on epidemiological and population characteristics by investigating their dependence on a range of parameter values. Finally, we study infection with two strains of influenza, inspired by observations, and show a counter-intuitive result for the effect of inoculation against the strain that leads to the first wave of infection.

## Introduction

Infectious diseases have been and continue to be a major public-health problem [1] affecting the quality of life and reducing life expectancy of human populations with attendant negative, socio-economic effects [2]. The emergence of new diseases and the re-emergence of mutant strains have added to their enormous negative impact. The control of influenza A virus, a major international health concern [3] poses a scientific challenge at many levels. According to the CDC, on average, about 8% of the U.S. population falls ill from flu each season, with a range of between 3% and 11%, depending on the season [4]. The problem of influenza spread and proliferation spans length scales from the sub-cellular to the national and time scales from seconds to decades. There are many virus strains that display different pathogenic behaviors and elicit different immune responses at the sub-cellular and cellular scales [5]: the outcome of the infection is determined by the spatio-temporal dynamics of the competition between the propensity of the virus to spread, the host response to eliminate the pathogen, and their mutual antagonism.

There is a long history of mathematical modeling of infectious diseases starting from the work of Daniel Bernoulli in the eighteenth century studying the efficacy of small pox vaccination of healthy people. Many pioneering deterministic studies were carried out in the early years of the twentieth century, for example, by Ross and co-coworkers, and Lotka, especially on malaria. A significant milestone was the work of Kermack and McKendrick [6] who made a comprehensive study showing the existence of a threshold population size below which an outbreak does not occur. Several books and reviews provide a comprehensive introduction to recent work [7, 8]. Our paper reports results of numerical simulations of a stochastic agent-based model of the spread of influenza A over the course of a season and an analysis of the behavior of the model as population size, interconnectivity and immune strength are varied.

Numerous works have appeared on how agent-based models and stochastic models have been applied to infectious diseases and their implications for health [9, 10]. A simple version of the original mathematical model developed by Kermack and McKendrick known as the Susceptible-Infectious-Recovered (SIR) model has been used extensively and forms the basis of many investigations, including ours. Stochastic modeling was subsequently initiated by the pioneering work of Bartlett [11] and Bailey [12] and others. Rvachev conducted one of the earliest studies of spatial-temporal model of influenza and applied it to spread in the Soviet Union [13]. There are many models that have used stochastic simulation methods applied to metapopulation models and agent-based models to investigate the epidemiology of influenza and other infectious diseases. Watts et al. [14] introduce and study a class of meta-population models with a hierarchy of successively larger domains with mixing, finding behavior with qualitative agreement with features extracted from data. Epidemic processes have been studied in a metapopulation approach with different flows based on coarse-grained empirical data in the air transportation network [15]. For example, the impact of different strategies of intervention, random vs. targeted, on the spread of epidemics in a population, separated into subgroups in spatially discrete patches connected by migration pathways have been studied [16].

Guo et al. [17] combine a heterogeneous graph modeling approach to build a social network for a specific region (Forsythe County, North Carolina) with an Agent-Based Model and found good agreement with clinical data. Fox et al. [18] use a stochastic model to argue that seasonal flu leaves a short refractory period of heterosubtypic immunity which impedes the emergence of novel flu viruses and used it explain the seasonality of the emergence of pandemic viruses. Computationally efficient implementations of household models have been described by Ross et al. [7], while Roche et al. [19] develop and validate an Individual-Based Model (IBM) and address the ecology, epidemiology, and evolution of influenza A viruses. Longini et al. studied a stochastic influenza simulation model for rural Southeast Asia and determined the effectiveness of targeted antiviral strategies for containing an emerging influenza strain at the source as a function of the input reproduction ratio [20]. It is known from these and other studies that the network structure of contacts determines the spreading of infection.

The data reported by the CDC show that the number of influenza-like illnesses (ILI) measured by hospitalizations or outpatient visits can show more than one peak within a season in some years at the national [21], regional [22], and city [23] levels. There can be multiple reasons for such recurrences in the data. In this paper, we define and study a simple agent-based epidemiological model, with probabilistic dynamical rules, with the goal of describing a mechanism that can lead to recurrences within a flu season; we investigate how varying the topology of the network structure of contacts known to determine the spread of infection (see above) affects the occurrence of recurrences. We analyze, inter alia, the sample-to-sample variability due to stochastic fluctuations and their statistical properties. The antecedents of our model lie in the early work of Bartlett who used ordinary differential equations and numerical simulations to show that recurrences can occur in measles epidemics on the scale of years when immigration is included in the model. Emphasis is on the behavior of the spread of influenza infection during the course of one flu season, and we show that recurrences that are seen in the data can occur in the simulations for certain network structures of contacts and values of rate constants.

We study the model in cases where the randomly occurring recurrences are well-defined to investigate their statistical properties. We use these results to study distributions of times at which the first peak occurs and the time intervals between the first and second peaks that underlines the highly stochastic behavior of the model. As reported by the CDC, as well as the city of Columbus, Ohio [23, 24], the 2018–19 influenza season had two waves of influenza A activity of similar magnitude: A(H1N1), a pandemic strain first, followed by a seasonal virus A(H3N2). Motivated by this observation, we have used our model to study two strains of infection, seasonal and pandemic, with a vaccine effective only for the pandemic, and show that the intensity of the second wave of seasonal infection can be lower with lower inoculation rate for the pandemic virus.

The rest of the paper is organized as follows: in the Model and Methods section, we describe our model based on the SEIR paradigm with a number of agents that are divided into cohorts with strong transmissibility within, and weak connections between successive cohorts. The assumptions made that render the model parsimonious and tractable, yet describe behavior of interest, are specified. In the Results section, we first report the results of an ODE model of two groups or cohorts for weak coupling between the groups that provides a point of comparison for the ABM simulations. We present simulation results for the spread of infection: the dynamics of the infected number, reproduction number, and features of probability distributions that characterize the stochastic behavior and how these vary with the number of inter-cohort connections and strengths. Results obtained from our model for the case of a two-strain infection are then discussed. Our results and conclusions are summarized briefly in the Conclusions. Supplementary material provides more detailed support for various statements in the main text.

## Model and methods

In early, seminal work in the context of measles epidemics, Bartlett showed the existence of a critical population size below which the infection dies off and the occurrence of recurrent behavior if there is immigration providing a supply of susceptible individuals [25]. He argues this by examining the Hamer-Soper model [26], a model with *s* susceptible agents and *i* infectious agents, which evolves stochastically in time. Three possible “transitions” can occur per unit time with specified rates: infection, defined by *s* → *s* − 1 and *i* → *i* + 1 at a rate λ *is*, recovery, defined by infectious agents recovering *i* → *i* − 1 and becoming permanently immune at a rate *μi*, and immigration, in which the susceptible agents *s* → *s* + 1 at a rate *ϵ*. The first two correspond to the transitions in the SIR model and as we discuss below, the population structure of our model mimics an immigration-like term.

Bartlett [11] studied the stochastic differential equations that describe these transitions and derived the deterministic approximation that gives rise to the standard equations.

$$\begin{array}{c} \dot{s}=\left(\epsilon -\lambda i\right)s,\;\dot{i}=\left(\lambda s-\nu \right)i. \end{array}$$ (1)

He showed that these equations lead to damped oscillations with a period that varies as $1/\sqrt{\epsilon }.$ Through small simulations, he demonstrated that the stochastic formulation of the model also leads to oscillations; thus, Bartlett obtained recurrences by introducing a source of susceptible individuals through immigration. We seek to explain recurrences (peaks in the reported infection numbers) on a very different time scale, within one flu season (30 weeks compared to the interval of 70 weeks or more for measles [27]) and study the agent-based model described in detail below. The population structure of the model consists of two or more cohorts that are strongly connected within, but with weak transmission (slower time scales) between cohorts. If only the first cohort is infected initially, the slow spread of infection to the second cohort effectively introduces additional susceptible individuals. This basic feature underlies our agent-based model that displays recurrent behavior of infected number with substantial stochasticity.

We present the details of our stochastic agent-based (individual-based) model that allows us to investigate the behavior of the dynamics of infection in a population as a function of the virulence of the infection, level of inoculation, details of the contact between individuals, and number of virus strains.

We define a set of agents (or individuals), each of whom can be in one of four states that correspond to the epidemiological SEIR model: susceptible (S), exposed (E), infectious (I), and recovered (R). Since it is known from data for the pathogenesis of the influenza virus that there is an incubation period corresponding to the non-contagious, exposed state [28], we use the SEIR model. The system evolves in time according to probabilistic rules with agents making transitions between states with rate parameters chosen to reflect the known features of influenza A dynamics. The primary quantity of interest is the number of infected agents as a function of time, both in individual samples, as well as the trends in the mean calculated over an ensemble of simulations. These depend on the epidemiological parameters enumerated above, as well as the population structure of the model. Individual sample dynamics correspond to data over a season in a region and display fluctuations with potentially important public health consequences.

The time evolution is assumed to occur in discrete time, with a time step of 1 day, which is appropriate for describing influenza infection in a group of individuals in a single season. We simulate for a time of one season: influenza strains mutate on the time scale of a year [29], particular influenza strains tend to dominate in each season, and which strain is a threat varies from year to year. Due to the difficulty in modeling the antigenic drifts on longer time scales, as well as the coarse-grained description of the infection in our model, we limit our simulations to a single season. In addition, it is common for municipalities to collect data on influenza infections for this time period, as is the case for our location. We choose the time step to be one day because available immunological data which details the course of influenza infection for individuals is on the scale of days to weeks [24]. One day is also roughly the time scale on which a person would be able to characterize their personal immunological state, and is thus a reasonable time unit.

Simulations are initialized with *N* agents, placed into *M* cohorts. Connections between agents are organized into a matrix, *C*, termed the contact matrix, whose elements *C*_*ij*_ describe the strength of the infectious contact between agent *i* and agent *j* when one of them is infectious and the other susceptible. First, connections within cohorts are specified. Each agent in a cohort is connected to *k* other random agents within their cohort. In graph-theoretic terminology, each agent is a vertex with degree *k* in the connection graph.

**Fig 1:**
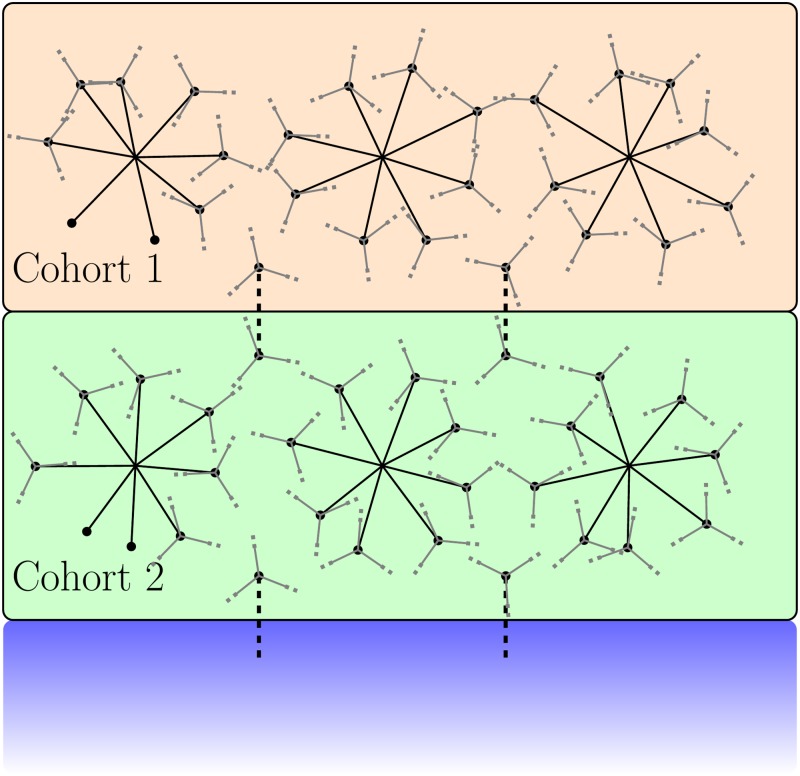
Schematic for the population structure of the model. Within each cohort, each agent is connected to a fixed number of randomly chosen agents. Successive cohorts are connected through relatively few spanning connections between agents in the two cohorts. The inter-cohort connections are shown dashed, representing a weak connection value compared to the intra-cohort connections. There are no connections between agents in non-adjacent cohorts. The blue box represents further cohorts which are modeled the same way as the first two. Specific values and details of the structure are given in Table 1 and in the text of the Model section.

Next, connections between successive cohorts (cohort *J* to *J* + 1 for *J* = 1, ….*M* − 1) are defined as follows. *k*_*bet*_ agents are chosen randomly from cohort *J* and each is connected *k*_*inter*_ agents in cohort *J* + 1. We used *k*_*bet*_ = 25 and *k*_*intra*_ = 1. The intra-cohort connections are always given the same value *C*_0_ (for explicit values, see Table 1). The inter-cohort connections are chosen to be weak in comparison, and fewer in number. The inter-cohort connection strength is taken to be *C*_0_/10. This structure defines a hierarchical model in which infections generally spread within cohorts, with infections between cohorts happening rarely. This separation leads to model behavior with peaks in the infected number and which is stochastic with respect to individual simulations. A schematic of this contact structure is given in Fig 1.

**Table 1:** Table of parameters used in ODE model and ABM.

Parameter symbol	Meaning	Value	Reference
*κ*	Infection Time-scale	0.5 days	[30]
I	Time to contagious	I∼U[1,4] days	[24]
R	Time to recover	R∼U[1,3] days	[24]
*C*_*ij*_	Intra-cohort contact strength	0.16	Adjusted
$C_{ij}^{\prime }$	Inter-cohort contact strength	0.016	Adjusted
*V*	Individual virulency	V∼U[0,3]	Free parameter
*I*	Individual immunological profile	I∼U[0,4]	Varied
*N*	Number of agents	6000	Varied
*E*_0_	Initial infection size	0.005 ⋅ N	
*k*_*intra*_	Intra-cohort connection degree	16	[14]
*k*_*inter*_	Inter-cohort connection degree	1	Varied
*k*_*bet*_	Inter-cohort connection number	25	Varied
*β*_*ii*_	Intra-cohort ODE infection rate	2 day^−1^	
*β*_*ij*_	Inter-cohort ODE infection rate	*β*_*ii*_/200	
*ν*	ODE recovery time	2 days	
*σ*	ODE exposed transition time	2 days	

**Fig 2:**
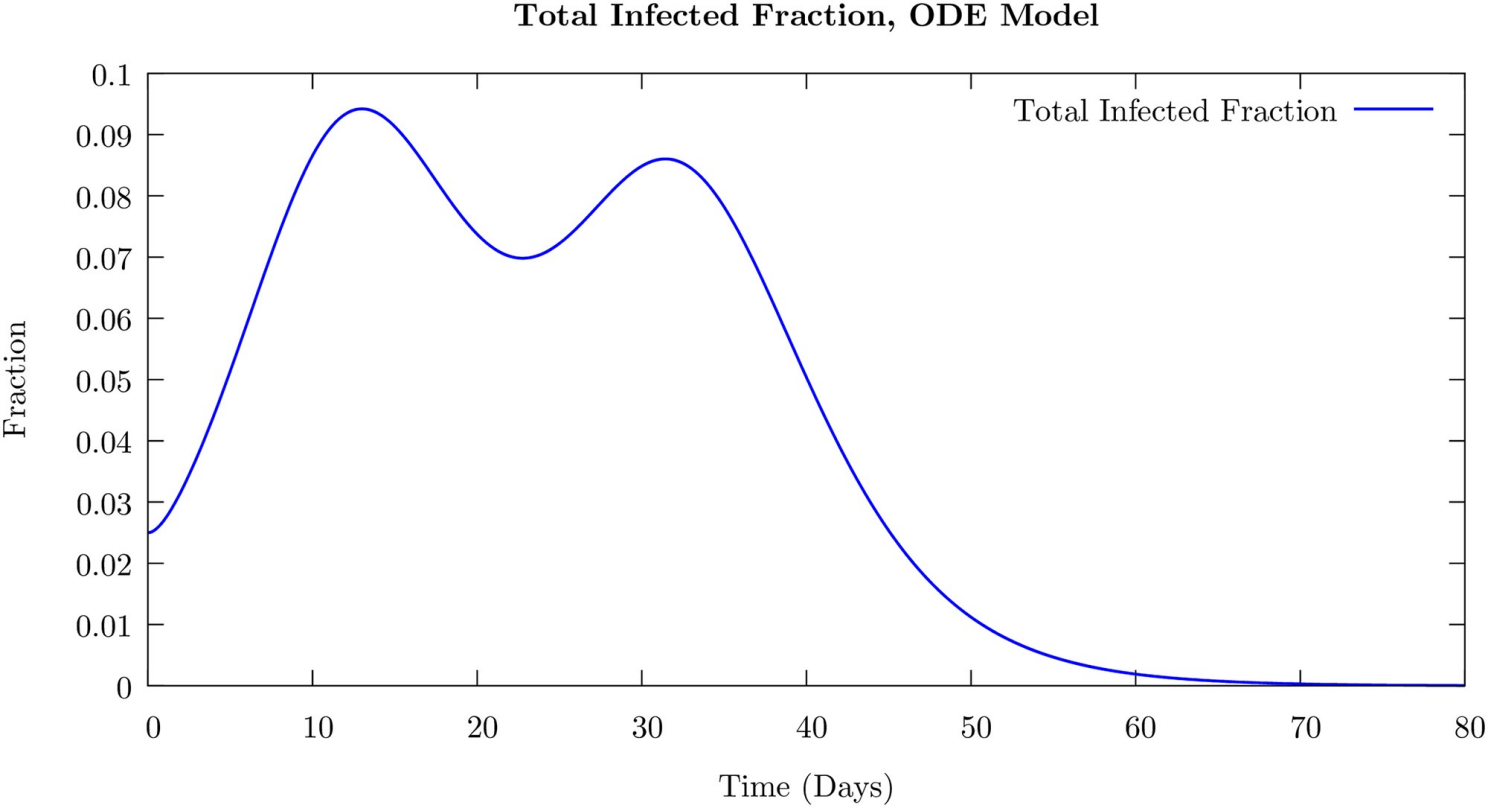
Total infected (sum of exposed and infectious agents) number as a function of time for two-cohort SEIR model defined in Eq 3. Here, *β*_11_ = *β*_22_ = 2, and *β*_12_ = *β*_21_ = 0.01, and the recovery rate *ν* is 0.10. The separation in infection timescale between intra-cohort spread and inter-cohort spread leads to two distinct peaks in the infected number. This peak separation decreases if the infection rates are made closer in value, as well if the recovery rate is made too rapid. This behavior is compared with the mean behavior of the infected number in the ABM later in the text.

**Fig 3:**
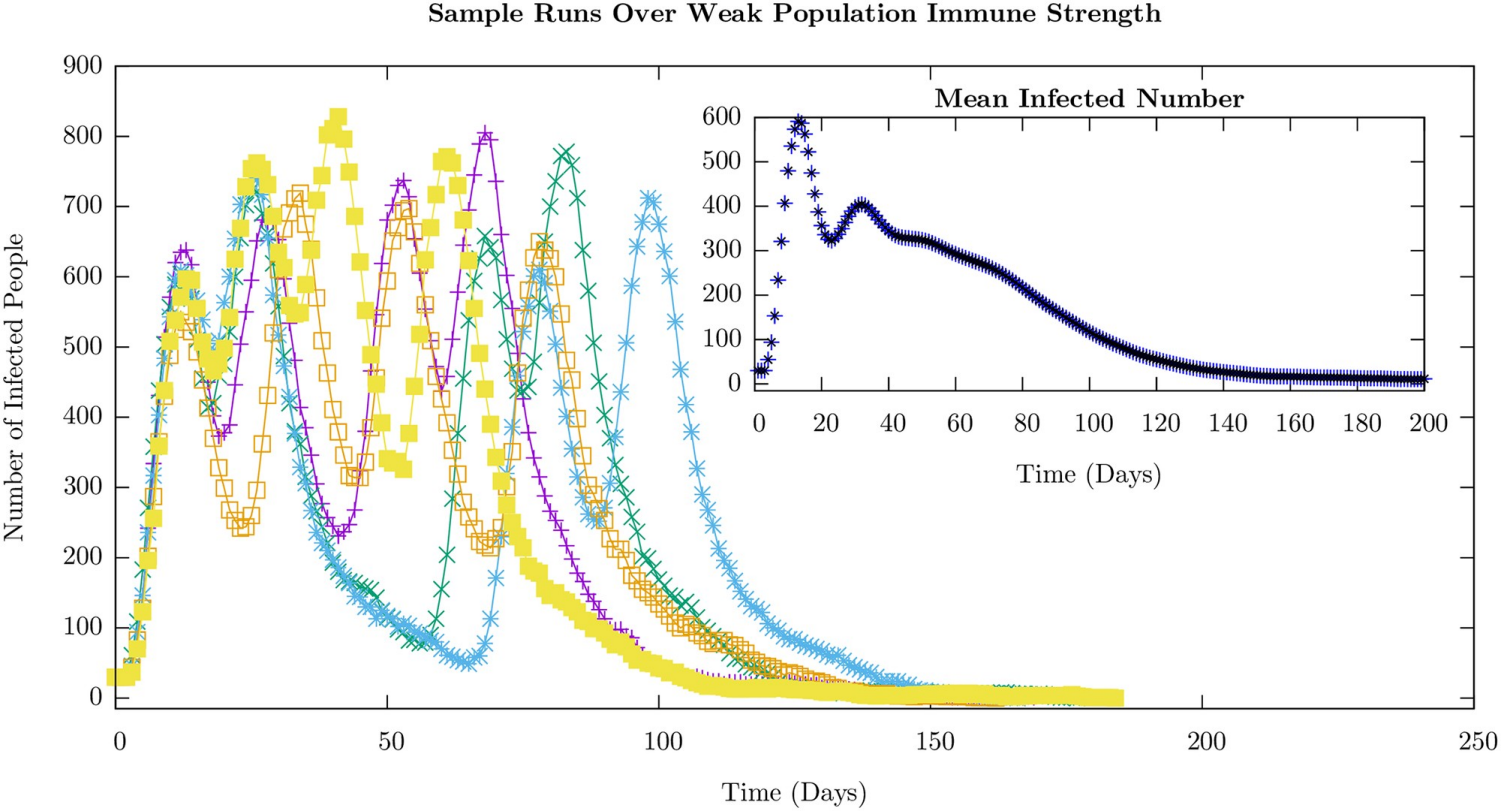
ABM results for the infected (the sum of exposed and infectious) agent number time series, with *I*_*i*_ ∈ [0, 4], for a population with weak immune strength corresponding to no immunization prior to the onset of the infection. Individual sample runs are shown along with the mean infected number, averaged over 10,000 runs, in the inset. This level of immunity leads to time scales for the rate of infection transmission between individuals comparable to or faster to the time duration before which an infectious individual recovers. However, the inter-cohort spread, through the weak, intermediate connections, is slow and the position of the peaks in the succeeding cohorts successively connected varies from run to run. The statistics of where the first peak occurs and the time interval between the first and second peaks are shown in Figs 5(a) and 7. The other parameters used in the simulation given in Table 1.

**Fig 4:**
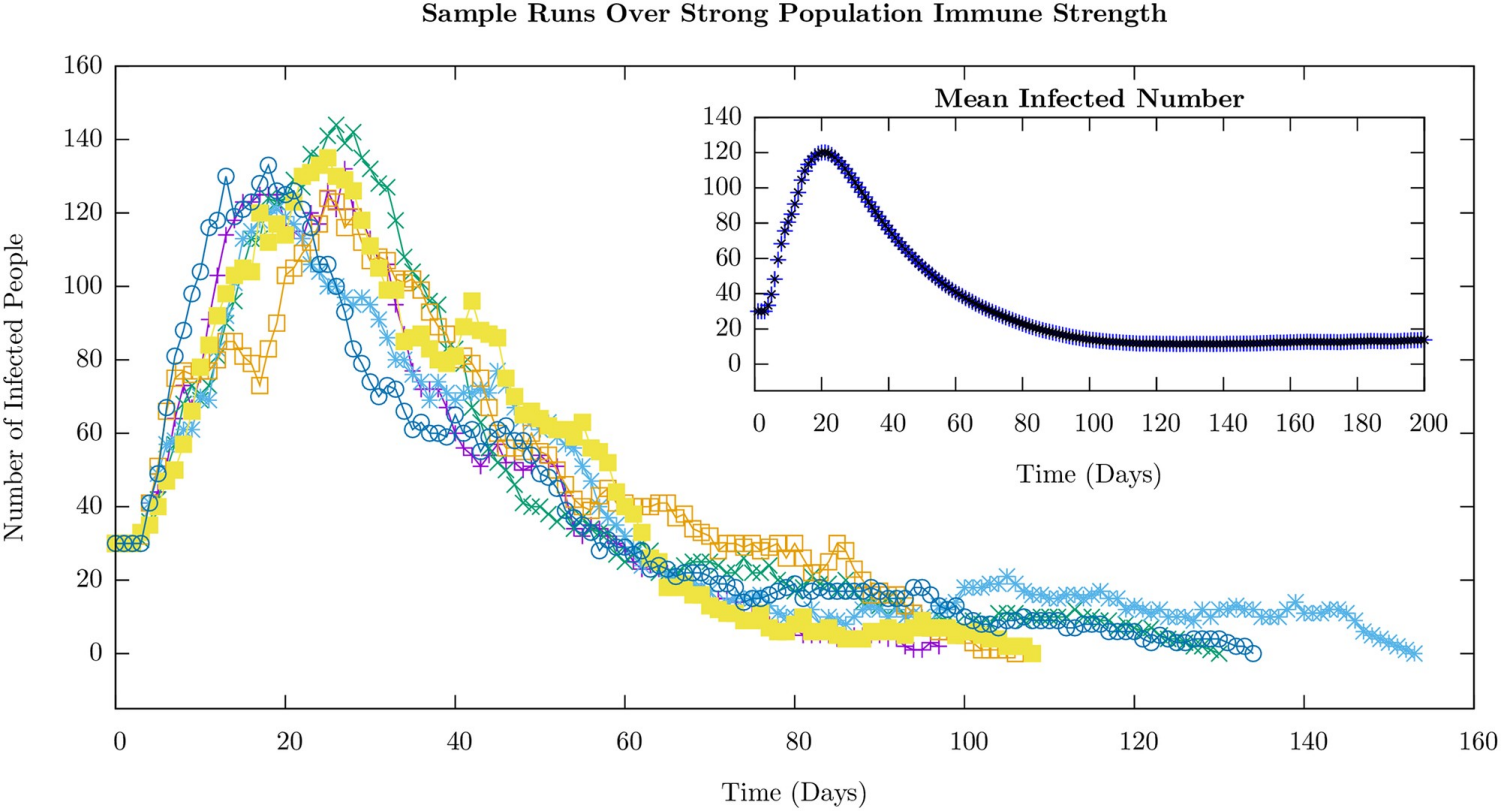
Plots corresponding to Fig 3 for the infected (the sum of exposed and infectious) agent number time series, with *I*_*i*_ ∈ [2, 6], a case of strong immune strength corresponding to immunization prior to the onset of the infection. Individual sample runs are shown with the ensemble average taken over 10,000 runs in the inset. This level of immunity leads to slow time scales for the rate of infection transmission between individuals compared to the duration before which an infectious individual recovers. The inter-cohort spread, through the weak, intermediate connections, is slower still and so we see an overall decay of the number of infectious agents. One sees smaller peaks punctuating the overall decline occurring randomly in time. The statistics of where the first peak occurs is shown in Fig 5(b). The other parameters used in the simulation are given in Table 1.

Parameters used for ABM results given in Figs 3 and 4, and ODE results given in Figs 2 and 8. Parameters listed as “varied” are varied to investigate the effect on the results presented. Parameters listed as “adjusted” are chosen based on the timescales given by observed data. ODE parameters were set to reproduce the ABM behavior qualitatively.

This style of hierarchical population structure has previously been observed across different regions and has been utilized in several modeling studies; observations of networks with densely connected cohorts and weak inter-cohort connections arose in STD studies in which individual agents either travel between cities or are separated socially from each other [31–33]. Combining modeling and real survey data for the study of influenza, Longini et al. [20] utilized a hierarchical population to model influenza spread through close groups (families, schools, workplaces) and casual groups (markets and shops). The strength of connection between these groups was reduced as a function of geographical distance according to a study conducted in the region modeled. While a model in which the agents may move between cohorts, Watts et al. [14] also define a many tiered population structure in which recurrent influenza infections are shown to occur. The closely connected contexts in their model are roughly the same size as clusters within our cohorts. This style of structure was also used to model SARS spread in Hong Kong by Riley et al. [34]. There, the hierarchical structure once again arose from modeling spatial distance between weakly interacting groups. While different forms of the contact matrix have been used, the values of the elements are not known. The structure of *C*_*ij*_ in this work is similar to that in previous models while the details differ. We have examined the dependence of our results on features of the topology, such as the inter-cohort connectivity.

Now, we describe the rules that govern the transitions of the agents between the states at each time step. At the beginning of the season, every agent is in either of two states. Most are in the susceptible state, and a small fraction are in the exposed state; the latter can be viewed as the initial infection fraction. Each exposed agent remains in the exposed state for a period I at the end of which the agent makes a transition to the infectious state. In our model, the duration of I over which the infection is latent is distributed according a uniform distribution between 1 and 4 days. Upon exiting the latent period, the exposed agent enters the infectious state. Infectious agents recover in R days after turning infectious (i.e. the agent undergoes a transition from the infectious to the recovered state) where R is uniformly distributed between 1 and 3 days. The distributions are within the ranges specified by the CDC as well as by clinical guidelines [24, 35]. There is no single generally applicable distribution for these times: for example, R will depend on the efficacy of the antiviral drug, the particular individual’s immunological profile, whether they were vaccinated or not, and the successful establishment of immunity by the vaccine.

These distributions depend on fine-grained details of immunology and virology that are currently unknown; the study of immunogenicity of vaccines is rudimentary [36, 37] and data on vaccination-induced antibody titers are sparse. We therefore choose a uniform distribution (given evidence for large variations among individuals in the references cited) for both R and I. One can justify this formally as follows: if one adopts the principle of maximum entropy, in the absence of information (other than the interval of variation), the distribution with the largest entropy should be chosen as the least-informative default. The uniform distribution is the maximum entropy distribution among all continuous distributions defined on a finite line.

It is possible to generalize this model substantially by allowing these distributions to vary as a function of time, infectivity, contact with other individuals, and many other possible effects. Our goal is to study how the behavior changes when rates are varied according to biological and epidemiological considerations, and it would be well-nigh impossible computationally to include such effects. Quantitative characterization of the parameters from real data is difficult because of the multiplicity of causes for observed effects.

Each susceptible agent is characterized by an immune strength that is determined by their immunological profile, whether they are vaccinated or not, and how effective the vaccine is [36]. We denote this by *I*_*i*_ for the *i*^*th*^ agent and the probability per day that a susceptible agent becomes exposed upon contact with an infectious agent decreases with increasing *I*_*i*_. This provides a simple way of including the effect of immunization in the model; the immune strength of an agent, *I*_*i*_, increases in value, resulting in an increase in the mean time to become exposed by an infectious agent. A fully effective vaccine would extend this mean time well beyond the duration of time that an agent remains infectious, thus insulating the susceptible agent from infection. Different values of the immune strength *I*_*i*_ can be interpreted as being partly due to vaccine efficacy. While it is clearly a drastic simplification to represent the immunological profile by a single number, as more data become available, more detailed characterizations can be used. The value for each agent is chosen from a uniform distribution due to lack of data on individual variation in immunological profile.

Each infectious agent *j* is characterized by a parameter, *V*_*j*_, which represents the infectivity of the individual *j* in the infectious state. It is clearly insufficient to characterize infectivity by a a single parameter since it encompasses the relative rate of viral shedding, personal hygiene, and many other parameters that vary from individual to individual and can be expected to be multi-dimensional. Each agents’ infectivity is drawn from a uniform distribution in the interests of parsimony and the lack of data characterizing variation in individual infectivities. Further, the distributions for *I*_*i*_ and *V*_*j*_ realistically vary with time, a complication that is ignored in our model. Information on the variation of the infectivity in time and the differences between individuals is not available. We can interpret the constant value assumed in the model as the time-averaged value and the individual variations as being encapsulated in the uniform distribution. This is consistent with our overall goal of parsing the effect of the variations of a few parameters on the behavior of a relatively simple model. Isolating the effects of specific dynamical behaviors will be computationally prohibitive if the number of parameters is increased.

An exposed agent *i* is simply characterized by the state (E) until they turn infectious at the end of the period $I_{i}$, chosen for that individual at the time of exposure from the distribution specified above. Susceptible agents are dynamically inert until their contact with infectious agents gives them a probabilistic rate to become exposed as described next. We define the probability per day for a susceptible person, *S*_*i*_, to become exposed as

$$\begin{array}{c} P\left(S_{i}\to E_{i}\right)=e^{-I_{i}}\sum_{j}\frac{\tanh(C_{ij}V_{j})}{\kappa J} \end{array}$$ (2)

where *C*_*ij*_ is the contact matrix connecting susceptible individual *i* to infectious individual *j*, *V*_*j*_ is the infectivity of individual *j*, *J* is the total number of infectious agents connected to the susceptible agent *i* under consideration, and *I*_*i*_ characterizes the immunological profile of susceptible individual *i*. Since the immune strength, *I*_*i*_, enters as an exponential, $e^{-I_{i}}$, increasing this value decreases the probability to infect, implying a longer mean time to become infected as discussed earlier in this section. The probability is non-zero only if there is at least one agent *j* for which *C*_*ij*_ > 0 (there is contact) and the agent is infectious (*V*_*j*_ > 0). The tunable parameter *κ* is time-independent, and sets the time scale on which infection is transmitted: it determines the range of values of *P*(*S*_*i*_ → *E*_*i*_). Due to the simplicity of how the immune strength enters the rate of infection, our results do not depend on the details of the distribution unless endowed with more structure, such as bimodality. The intra-cohort contact matrix elements are taken to be *C*_*ij*_ = 0.16 while the inter-cohort matrix elements are smaller: 0.016. *V*_*j*_ is in the range [0, 3], and *κ* is taken to be 0.5 days. Parameters are given in full in Table 1.

We comment on the choice of the hyperbolic tangent function. Clearly, the probability should increase with the number of contacts with infectious agents and their infectivities. Our choice makes this linearly proportional for small values of the argument of tanh(*C*_*ij*_ *V*_*j*_) and prevents it from exceeding unity. In practice, the argument rarely exceeds $\frac{1}{3}$ and it typically less than $\frac{1}{10}$. We have checked in several cases that the use of $\frac{x}{1+x}$ instead of tanh(*x*) makes very little difference to our results.

The implementation of the algorithm is straightforward. At any time *t*, one picks an individual *i* who is susceptible, and generates a random number uniformly distributed between 0 and 1. If it is less than *P*(*S*_*i*_ → *E*_*i*_), the individual *i* becomes exposed (the transition occurs). We choose three attributes that will be used in the future dynamics: a randomly chosen period $I_{i}$, after which the agent becomes infected, $R_{i}$, the period for which the agent remains infectious, and the infectious parameter *V*_*i*_ during the period of infectivity. These values are all chosen from distributions described above. If the random number we picked is larger than the probability to become exposed for agent *i*, the agent remains susceptible until the next day.

Once all susceptible agents in all the cohorts are checked to see if they will turn exposed or not, the remaining agents are checked for their possible transitions: exposed becoming infectious or infectious becoming recovered if the time elapsed after they become exposed or infectious exceeds their assigned durations {I} and {R}. Recovered agents do not participate in the dynamics. We then advance the time by one day, and repeat the procedure for a season, chosen to be 200 days.

The structure imposed on the model by various choices in *C* provides opportunities to analyze interesting behavior inherent to either the discrete nature of the model or the separation between groups of agents naturally afforded by various contact matrices.

The choice of different distributions for *I*_*i*_, *V*_*j*_, and the definition of the contact matrix *C* gives a great deal of flexibility once the model is implemented, allowing one to include information from analysis of data and to explore how different choices for the distribution influence how the epidemic spreads.

The code to simulate the model was written in C++, with data analysis performed in C++, *Mathematica 11.3* [38] and R, and visualization using gnuplot and R. During the analysis, it was necessary to find peaks in individual time series data. For this, a duplicate time series was created and smoothed using Wiener filtering [39]. This smoothing was necessary due to the natural stochasticity of individual runs of the model. This smoothed time series was then analyzed using FindPeaks in *Mathematica 11.3* [40]. The peaks were then mapped back to the original time series and checked for accuracy.

### Deterministic ODE model

Studies of deterministic models can provide insights into the behavior of the mean values of the dynamical variables in a stochastic model [41]; the latter provides information about fluctuations and sample-to-sample variability. In order to interpret the results of the mean values obtained from the simulation of our ABM, we present a model of ordinary differential equations (ODEs) that incorporates the cohort structure built into the ABM described above. The model is an SEIR model with just two cohorts for simplicity. The dynamical variables are *S*_*j*_(*t*), *E*_*j*_(*t*) and *I*_*j*_(*t*) (with *j* = 1, 2), which are respectively, the fraction of susceptible, exposed, and infectious individuals in cohort *j* at time *t*. The differential equations governing the time evolution are given below:

$$\begin{array}{c} \begin{array}{cc} \frac{dS_{1}}{dt} & =-\;\left(\beta_{11}I_{1}+\beta_{12}I_{2}\right)S_{1}\\ \frac{dS_{2}}{dt} & =-\;\left(\beta_{21}I_{1}+\beta_{22}I_{2}\right)S_{2}\\ \frac{dE_{1}}{dt} & =-\;\sigma E_{1}\left(t\right)+\;\left(\beta_{11}I_{1}+\beta_{12}I_{2}\right)S_{1}\\ \frac{dE_{2}}{dt} & =-\;\sigma E_{2}\left(t\right)+\;\left(\beta_{21}I_{1}+\beta_{22}I_{2}\right)S_{2}\\ \frac{dI_{1}}{dt} & =+\;\sigma E_{1}\left(t\right)-\;\nu I_{1}\left(t\right)\\ \frac{dI_{2}}{dt} & =+\;\sigma E_{2}\left(t\right)-\;\nu I_{2}\left(t\right) \end{array} \end{array}$$ (3)

The terms in the second equation describe, for example, the susceptible fraction in cohort 2 decreasing at a rate *β*_21_ because susceptible agents become exposed due to contact with the infectious fraction *I*_1_ in cohort 1, and also decreasing due to transmission within cohort 2 at a rate *β*_22_. There is a corresponding increase in the exposed fraction in cohort 2. The exposed fraction in a cohort turns over to the infectious fraction at a rate *σ*; the infectious fraction recovers at a rate *ν* and has no further effect on the dynamics.

We solve these equations numerically with *Mathematica* [38] using the *NDSolve* function. We note that the transition between different states of an agent occur in discrete time units of days in the ABM, while it occurs in continuous time with an exponential change in the ODE model. We report results for our choice of parameters for the ODE that are close to those used in the agent-based model.

## Results

### Recurrences in ODEs

We begin by presenting the results from the numerical solution of the ordinary differential equations describing a two-cohort SEIR model discussed in the Model and Methods section. The results capture the behavior of the mean value of the dynamical variables such as the infected fraction obtained from the stochastic simulation of the agent-based model discussed subsequently. The ODE solution also provides a computationally efficient way of finding parameter values that lead to different behaviors. Our agent-based model with weakly connected cohorts and probabilistic transition rates between different classes, in contrast, displays substantial sample-to-sample variability as discussed later.

The results of the simulation for the total infected number *I*(*t*) = *I*_1_(*t*) + *I*_2_(*t*) + *E*_1_(*t*) + *E*_2_(*t*) are presented in Fig 2. We choose the intra-cohort transmission rates *β*_11_ = *β*_22_ = 2 to be much larger than *β*_12_ = *β*_21_ = 0.01, the inter-cohort transmission rates; thus, the inter-cohort spread occurs on a much longer time scale than the spread within the cohort. The time for the exposed fraction to become infectious 1/*σ* = 2 days, and the recovery time of the infectious fraction 1/*ν* = 2 days are close to those in the agent-based model discussed above.

When only the first cohort is exposed initially, we find two peaks in the total infected fraction as a function of time as shown in Fig 2. We have normalized the fractions so that the total in each cohort is unity. In contrast, in the usual single cohort SEIR model with a small initial infection, there is only one peak in the infected number as a function of time.

As the separation of the infection time scales is reduced by increasing *β*_12_, the time difference between the two peak positions decreases and eventually, the peaks merge into just one peak. We note that introducing a time delay in the SIR model so that while the susceptible population fraction decreased instantaneously upon infection at time *t*, the infected numbers increase only after a delay *τ*, corresponding to the time when they are in the exposed state, leads to two peaks. See S1 Fig. This is consistent with the fact that sufficient time delays can induce oscillatory behavior in ordinary differential equations [42]. We note that the agent-based models incorporate such time delays in their definition, for example, corresponding to the time during which an individual is exposed but not infected.

### ABM results

We discuss the results of numerical simulations of the agent-based model described in the Model section. Despite the many drastic simplifications made in representing the details of the complex processes that occur on many different length and time scales, simulations of the model lead to insights into the dynamics of the spread of infection. The primary quantity of interest is the time evolution of the number of infected agents (the sum of the number of exposed and infectious agents at a given time) given a small number of initially exposed individuals (0.5%) in the first cohort. The ODE model results presented above demonstrates that in a deterministic system, we can find recurrent behavior; here, we study the stochastic behavior in the discrete ABM with a finite number of agents, a limited number of contacts, and probabilistic time evolution.

We investigate the model with *N* = 6000 agents divided into *M* = 4 cohorts. While we present selected results for other values of *N*, the number of agents was chosen so that an exploration of different parameters can be done expeditiously. Each agent has an intra-cohort, out-degree of 16, i.e., is connected to 16 randomly chosen agents inside the cohort. This out-degree value is consistent with various other population models [14, 17] which find the degree of connection tends to vary between 6 and 20, depending on the method of data analysis. Inter-cohort connections are chosen as follows: 25 agents in a cohort (2% of the cohort size) are randomly chosen to have one connection to an agent in the next cohort. The number of inter-cohort connections is varied in the simulations to examine the dependence of the behavior of the model on this number, and the results are displayed later in the section.

As explained in the previous section, a single parameter *I*_*i*_ characterizes an individual’s immune profile, consisting of various features: the immunogenicity and protective efficacy of the immune system which is enhanced by vaccination. We investigate two cases; one corresponds to a population with a “strong” immune profile that can be viewed as arising from immunization in which *I*_*i*_ is drawn from a uniform distribution of range 2–6. The second case is a population with “weak” immune profiles corresponding to no immunization or ineffective vaccines. In this case, the values of *I*_*i*_ range from 0 to 4. We note that the rate at which the infection spreads decreases exponentially with the immunization strength. The different parameter choices discussed in the Model section are given in Table 1.

We examine the temporal behavior of the number of infected agents, defined as the sum of the number of exposed agents and infectious agents at a given time. While it is difficult in practice to distinguish different states (exposed, symptomatic, asymptomatic but infected, etc. [43]), within the model, the number of infected agents can be easily determined and we report results for this number. We have verified that the number of agents in the infectious (i.e. contagious) state exhibits similar behavior.

We display the time course of the number of infected agents–on the average in the inset and for a few randomly chosen runs in the main body– for the weak and strong cases in Figs 3 and 4, respectively. The average value shows only a single peak in the case of strong immunity, and an additional shoulder in the weak immune strength case, which is very similar to the behavior displayed by the ODE model (see Fig 2). Nevertheless, the individual sample runs can show well-defined peaks in the weak immune strength case as is evident from the figure. The value of the infection number at the peaks are much higher, as would be expected, in the weak immune (low or no vaccination) case.

For the weak immune case (Fig 3), while the peaks are well-defined recurrences, their numbers and their positions appear random as can be seen even in the few runs. The statistics of the distribution of these peaks is presented later. In contrast, for the case in which the population possesses a strong immune profile, the secondary peaks are not well-defined. In fact, the time course of individual runs are characterized by strong short-time fluctuations that wash out the peaks. The short-time fluctuations are, however, much larger in the strong immune case compared to the weak immune case (compare individual runs in Figs 3 and 4). This can be ascribed to the intra-cohort dynamics being dominated by rare events as we explain now.

The immune strength *I*_*i*_ of the agents is uniformly distributed in the interval [0, 4] in the weak immune case and [2, 6] in the strong immune case. One consequence of the uniform increase of the immune strength by 2 units in the strong immune profile case is the reduced pool of susceptible agents that can be exposed in the course of the simulation that lasts a season of 200 days. In fact, *P*_*i*_ < 0.01 for *I*_*i*_ > 4. The stronger immune strength implies a decrease in the probability of a susceptible agent becoming exposed by a factor of roughly 7 (≈*e*^2^). The combination of fewer susceptible agents and lower probability for transitions means that new infections are relatively rare events. Since only a small number of new infectious occurs across the cohort in a short time interval, the fluctuations are large and this makes the peaks poorly defined in individual runs and smooths out the peaks on the average.

We present results for this set of parameters for which features such as peaks are better delineated so that their characteristics can be studied. When the parameters are varied over larger ranges, features such as the location of the peaks, their width and amplitude will be smeared out even in the case of weak immune strength. In the supplementary information, individual runs are given for several different sets of parameter variations which show how qualitative features of the peaks in the infected number time series change according to various ABM parameter choices.

We characterize the stochastic behavior of the model by computing probability distributions for different features of the time series for the infected numbers in the weak and strong cases: the distributions of the time at which the first peak occurs, the distribution of time intervals between the first and second peaks that is well-defined for the weak immune case, and the time at which the first susceptible agent in the second cohort becomes exposed.

**Fig 5:**
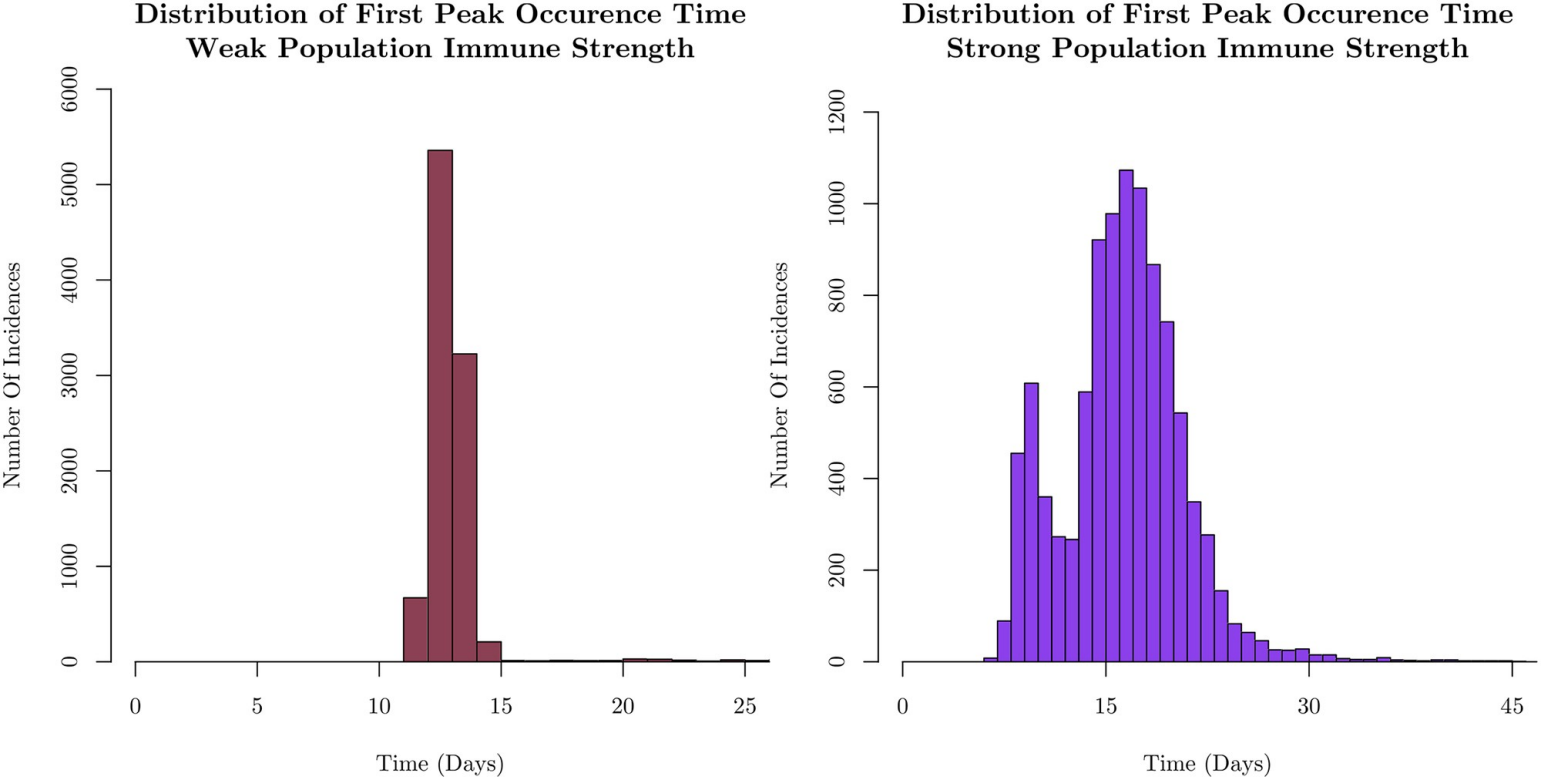
Distributions of the occurrence time of the first peak in the infected number. The results for a weak population immune strength corresponding to Fig 3 on the left, and for a strong population immune strength corresponding to Fig 4 on the right. For a population with weak immune strength, infection spreads rapidly throughout the initial cohort, leading to a narrow distribution of times for the peak. Rapid spread reduces variance in the timing of the peak due to the finite size of the cohort. Increasing the collective immune strength, as shown on the right, broadens the distribution reflecting the increased time to spread as well as the availability of fewer susceptible agents, leading to many pauses in infection spread throughout the cohort. The histograms each represent 10,000 sample simulations.

We show the distribution of the times at which the first peak occurs for weak and strong immune cases in Fig 5a and 5b. by averaging over 10,000 samples.

The distribution of first peak times in the weak immune case is narrowly peaked around 12 days, consistent with the few sample runs shown in Fig 3. On the other hand, it is much broader in the strong immune case. The introduction of a stronger immune response forces the infection to spread through relatively rare events as discussed earlier. We have monitored the probability for susceptible agents to be infected and become exposed, *P*(*S* → *E*); for the strong immune case, the values center around *P* ∼ 10^−3^. This means that infection spread is unlikely, even on the scale of the entire cohort. This manifests itself in the width of the distribution of first peak times, since each run will be driven mostly by statistically uncommon infections.

We explore another effect of events with low probability: we focus on the spread of the infection across cohorts; in particular, the time at which the first agent in the second cohort becomes exposed. This is a low-probability event because the number of agents in the second cohort who are connected to agents in the first cohort is small. We calculate the statistics for a situation in which we increase the number of inter-cohort connections for the weak immune case, and examine the first incidence of infection in the second cohort.

**Fig 6:**
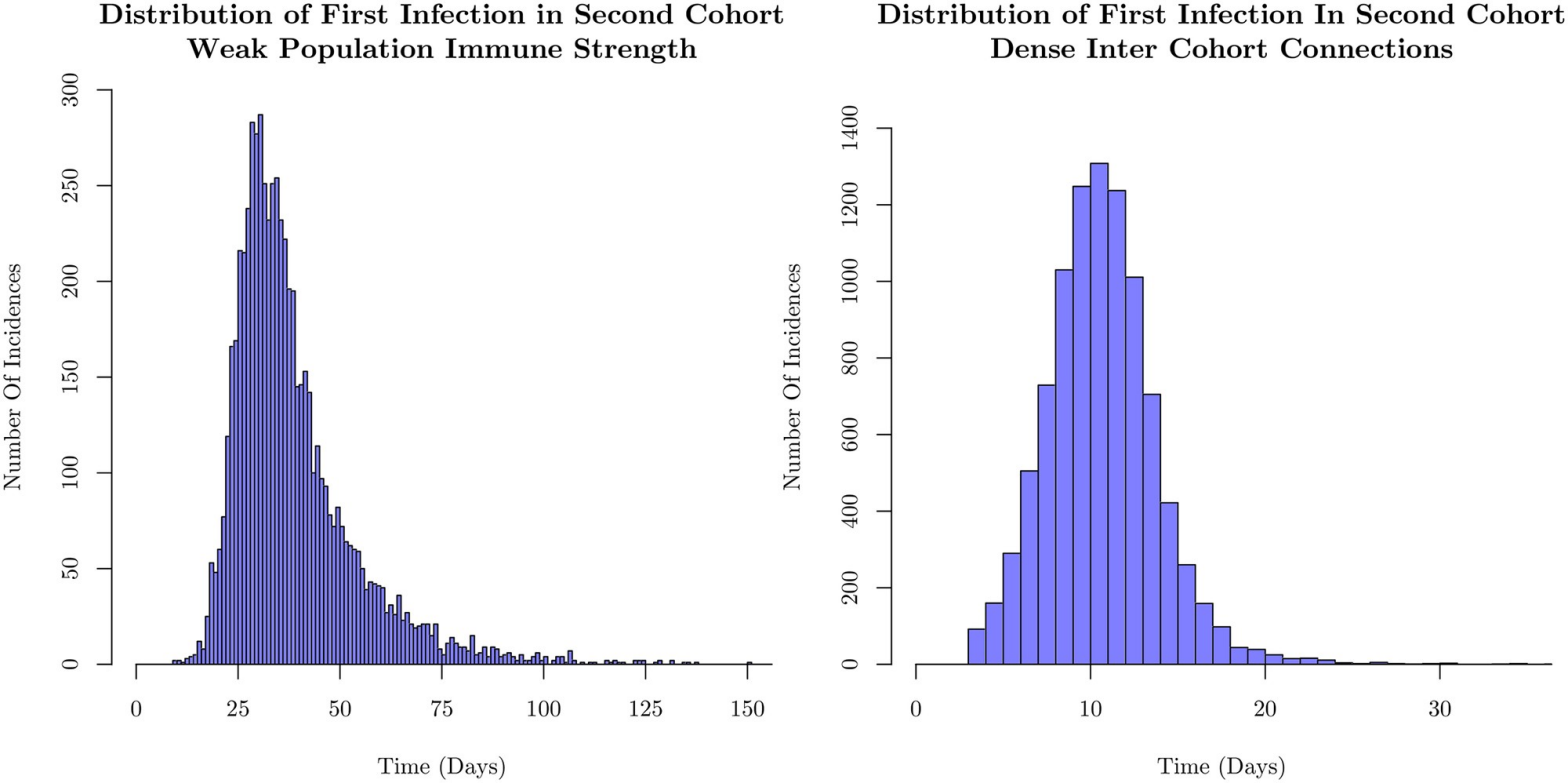
Distributions displaying the occurrence time of the first infection in the second cohort for weak immune strength. The figure on the left is for cohorts in which 2% of the agents are connected to the second cohort on the left, and on the right is for cohorts in which 10% of the agents are connected to the second cohort. The intra-cohort structure remains unchanged, and the immune strengths of the two cases are the same. The sparsely connected structure displays a very wide distribution of first infection times, despite rapid intra-cohort spread as shown in Fig 5. We show that with more inter-cohort connections, the distribution is both shifted earlier in time, as well as substantially narrowed. This demonstrates that much of the stochasticity in the model is driven by the weak inter-cohort connections that are relatively few in number slowing the spread through the population as discussed in the text. The histograms each represent 10,000 sample simulations.

The results are shown in Fig 6(a) and 6(b). The left histograms of Figs 5 and 6 correspond to the same set of simulations. We see that the distribution of times of first successful infection in the second cohort is very broad, despite the rapid spread through the first cohort. This is due to the relatively small number of weak inter-cohort connections. This bottleneck makes the dynamical behavior stochastic. Spread to the second cohort hinges on the small number of agents in the first cohort with connections to the second becoming infected. Typically, the small number of intra-cohort connections necessitates infection of a large fraction of the cohort before those few agents connected to the next cohort are infected. Since the probability of infection rarely rises above 0.1 for the parameters we have used, the spread is highly stochastic.

By increasing the number of inter-cohort connections, (from 2% of the cohort to 10%), in the right side of Fig 6, we see that the distribution of second cohort infection times both shifts earlier in time, as well as narrows substantially. This is consistent with the left panel of Fig 5, as the mean timing of the second cohort’s first infection is coincident with the timing of the mean first peak occurrence. The rapid infection spread statistically reaches both more agents which have connections to the second cohort, as well as reaches them sooner after initial infection. This underlines the importance of the separated nature of the contact structure for discernable peaks in the infected number; one important driver of well-separated peaks is the stochasticity inherent in spread between weakly connected subpopulations.

The distribution of first infection times in the second cohort in the strong immune case with few inter-cohort connections appears as broad as in the weak immune case. While this is surprising, it must be noted that a large fraction of the samples shows no infection in the second cohort within one season due to the low probability of the spread. Were the simulation time to be extended unnaturally, the distribution would be broader than in the weak immune case. Given that the distribution is constrained by the simulation time, we display it in the Supplementary section for completeness. See S6 Fig.

Another feature which characterizes the stochastic infection spread is the timing of the second peak of infection. Understanding the probability and the time of additional increases in the infection after the first peak, or recurrences, is of practical importance from a public health point of view [44]. As stated in the Methods section, peaks were found using FindPeak in *Mathematica 11.3*, after applying a Wiener filter for basic smoothing of the time series.

**Fig 7:**
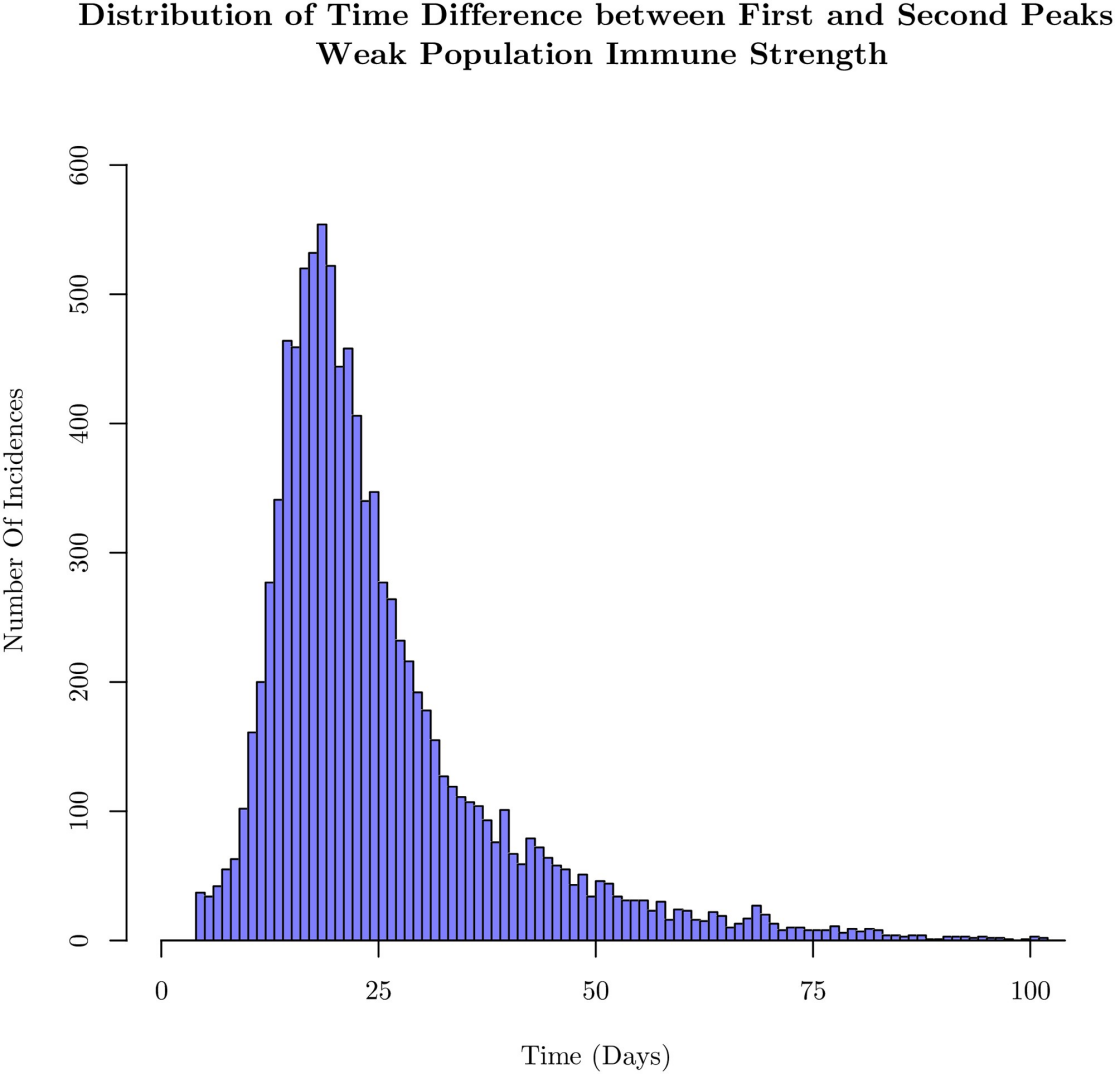
Histogram of the time between the first and second peaks in the time course of the number of infected (exposed and infectious) agents. Analysis is done for the same parameters as used in Fig 3. With a weak collective immune strength, variability in the contact structure and individual immune strength causes wide fluctuations in the time course of infection events. The histogram is constructed out of 10,000 sample simulations.

Despite limiting the variability of various parameters in cohorts and confining our attention to the stationary distributions for immune strengths and infectivities, there is considerable variation in the time interval between the first and second wave of infection in this model in the weak immune case. The histogram for the time differences is shown for weak immune system cases in Fig 7, and is broad even though the first peak occurs over a narrow range of times as seen in Fig 3. The appearance of the second peak is dominated by the weak inter-cohort connections as emphasized earlier. In the case of the strong immune profile, since it is difficult to identify peaks, we have not emphasized the features of the distribution, but have presented the result in the supplementary section. In S2 and S3 Figs, we show histograms of the number of peaks identified by our scheme: more than half of the samples in the strongly immune case have only one peak while more than three-quarters of the samples have more than one peak in the weakly immune case. This can be understood since it takes longer for the first cohort to have many infections in the case of a strong immune defense as seen in Fig 4. Given the bottleneck for the infection to spread to the second cohort, the second peak does not occur on the time scale of a season, the simulation time.

**Fig 8:**
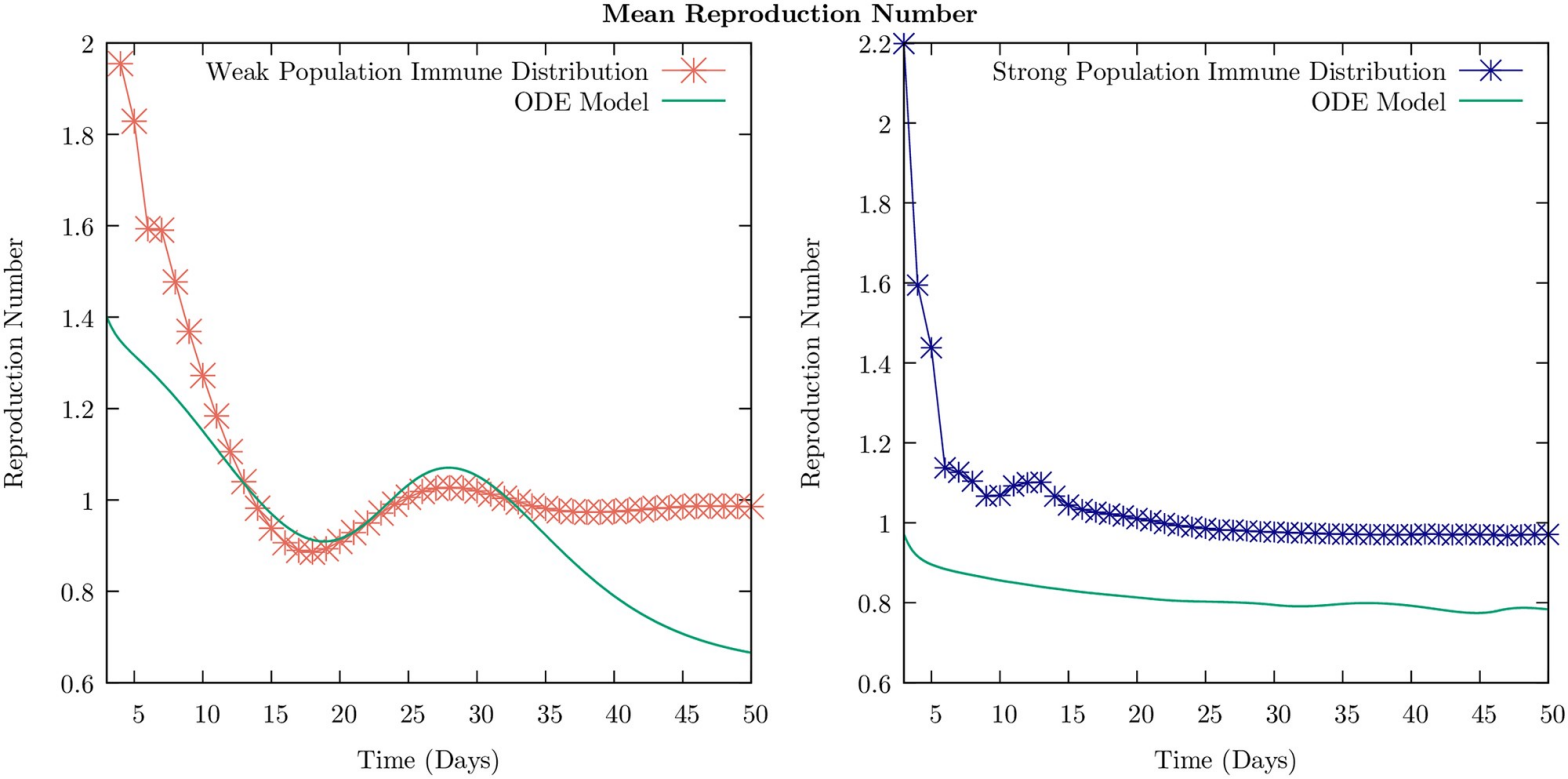
Mean reproduction number as a function of time for the weak and strong immune population distributions described in Figs 3 and 4, respectively. Means were calculated over 10,000 sample simulations, and are plotted as points with lines interpolated between them alongside the reproduction number defined for the two-cohort ODE SEIR model in Eq 4. Note that the ABM simulations were for a four-cohort structure, accounting for some differences between the two traces. We see that the effect of a global increase in the immune profile of the population is a suppression of (a) the initial spread of the disease, seen through a reduction in the reproduction number, and (b) of recurrent infection waves, seen through the elimination of the second interval of time where *R* > 1.

We discuss the behavior of the reproductive number as a function of time in the ABM and compare it with the result for the ODE model. We define the (instantaneous) reproductive number as the number of susceptible agents that an infectious individual infects during the time before recovery averaged over the sample. We have evaluated the reproductive number as a function of time in the ABM as follows: we count the change in the number of infectious people from one day to the next. We divide this number by the original number of infectious people from the previous day. This is an attempt to imitate the reproduction number one would calculate from field data, in which only the people who are visibly symptomatic are likely to report to a hospital. We relate our calculated quantity to the reproduction number simply as $1+\frac{\Delta I}{I}=R_{0}$. We calculate this quantity over 10,000 individual samples, and average the value at each time point. This ensemble average is plotted as a function of time, for both the strong and weak immune cases in Fig 8.

In the deterministic ODE model, we can define the instantaneous reproductive number as follows. The fraction that is newly infected in an infinitesimal interval *dt* in the two cohorts is *dI*_1_ + *dI*_2_ = (*dt*[*σ*(*E*_1_(*t*) + *E*_2_(*t*))] from Eq 3 that define the model. The reproductive number is

$$\begin{array}{c} R_{0}=\frac{1}{\nu }\frac{1}{I_{1}+I_{2}}\;(\frac{dI_{1}}{dt}\;+\;\frac{dI_{2}}{dt}) \end{array}$$ (4)

i.e., The fraction that is newly infected in the recovery time 1/*ν* per infected fraction *I*_1_ + *I*_2_. This is just the fraction of the susceptibles that become exposed and corresponds to the ABM calculation.

To understand this mathematically, we invoke the quasi-steady state approximation that is used for deriving the Michaelis-Menten equations for enzyme kinetics [45]. If we assert that the exposed fractions *E*_1_ and *E*_2_ are approximately in steady state, we can set $\frac{dE_{1}}{dt}$ and $\frac{dE_{2}}{dt}$ to zero (i.e., it is in a quasi-steady state and not changing in time), we can deduce that *σE*_1_ = *β*_11_ *I*_1_ *S*_1_ + *β*_12_ *I*_2_ *S*_1_ and similarly for *dE*_2_/*dt*. The result is shown in Fig 8 and is in good qualitative agreement with the ABM result.

### Analysis of results

We now examine how various features of the time series of infection, in particular, the occurrence of recurrences and the characteristics of the distributions that we have studied change when the model is altered, for example, by varying the population size and the structure of the contact matrix.

First, we analyze how the model results depend on the total size of the population. This is an important consideration both for application to real data, as well as computational considerations. For our analysis, an agent number higher than 8,000 considerably slowed computational speed. Therefore, a question of interest is whether qualitative features of the model remain approximately unchanged as the total population number is increased.

**Fig 9:**
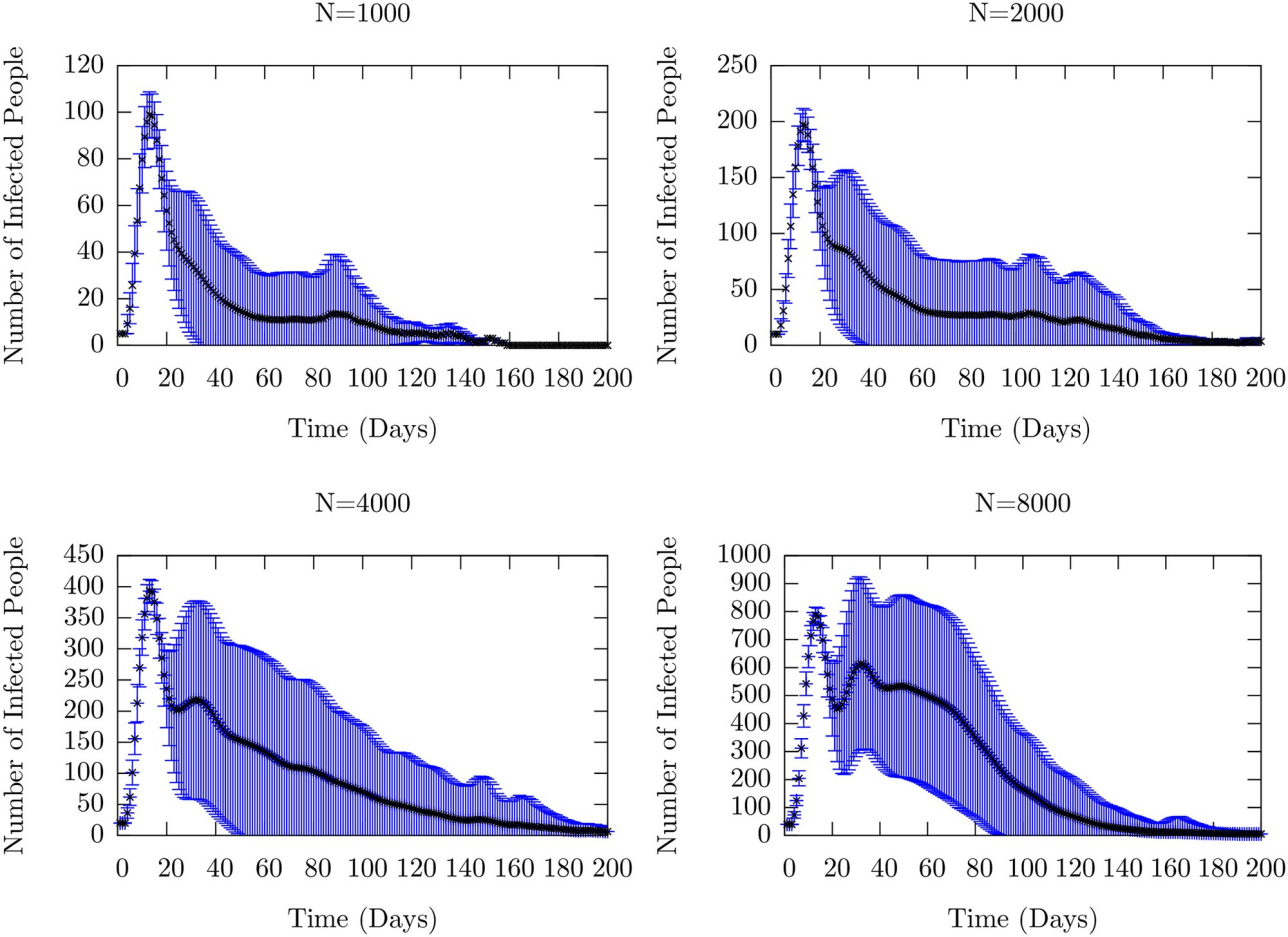
Mean number of infected (the sum of exposed and infectious) people as a function of time for four different total population sizes, all with the parameters given in Table 1. The black dots represent the ensemble average taken at each time point, with the error bars representing the ensemble standard deviations. Individual runs for each of these setups are shown in S7 Fig. We observe the increasing stability of recurrences as the population grows through the the second peak which arises as *N* is increased. For low values of *N*, (1000 and 2000), stochastic population collapse occurs frequently enough that there is only one well-defined peak due to the initial infection. The averages are taken over 10,000 individual realizations.

In Fig 9, we show the mean number of infected (the sum of exposed and infectious) agents as a function of time, averaged over 10,000 samples, for *N* varying from 1000 to 8000 for the model with a weak immune profile. An important feature of the plots is the stochastic instability at smaller values of *N*. Sample runs which contributed to these means are shown in S7 Fig. We see the peak caused by the initial infection, and a shoulder that becomes progressively better defined as we increase *N*, becoming a discernible second peak at *N* = 8000. For lower values of *N*, the number of infectious people can easily fluctuate down to 0, prematurely ending the simulation on that sample. This can be seen in the Supplementary Figure. With the introduction of larger numbers of agents, a stochastic fluctuation is less likely to collapse the run, leading to more opportunities for spread, and thus a recurrence of infection.

Thus, there is a stochastic suppression of the spread; such extinctions are known to occur in ecological models due to demographic stochasticity that leads to a downward trend for small population sizes, as well as in biological models [46, 47]. Recurrences occur and are relatively stable for values of *N* greater than 4000, continuing up through 20,000 individuals as we have checked. See S4 Fig for sample runs from a population size of 20,000. It is computationally prohibitive to perform a large number of samples.

The number and timing of the recurrence peaks remain random since we have kept the number of inter-cohort connections weak and few in number. This leads naturally to the question of the dependence of the behavior of the model on the inter-cohort and intra-cohort transmission probabilities that we examine next.

**Fig 10:**
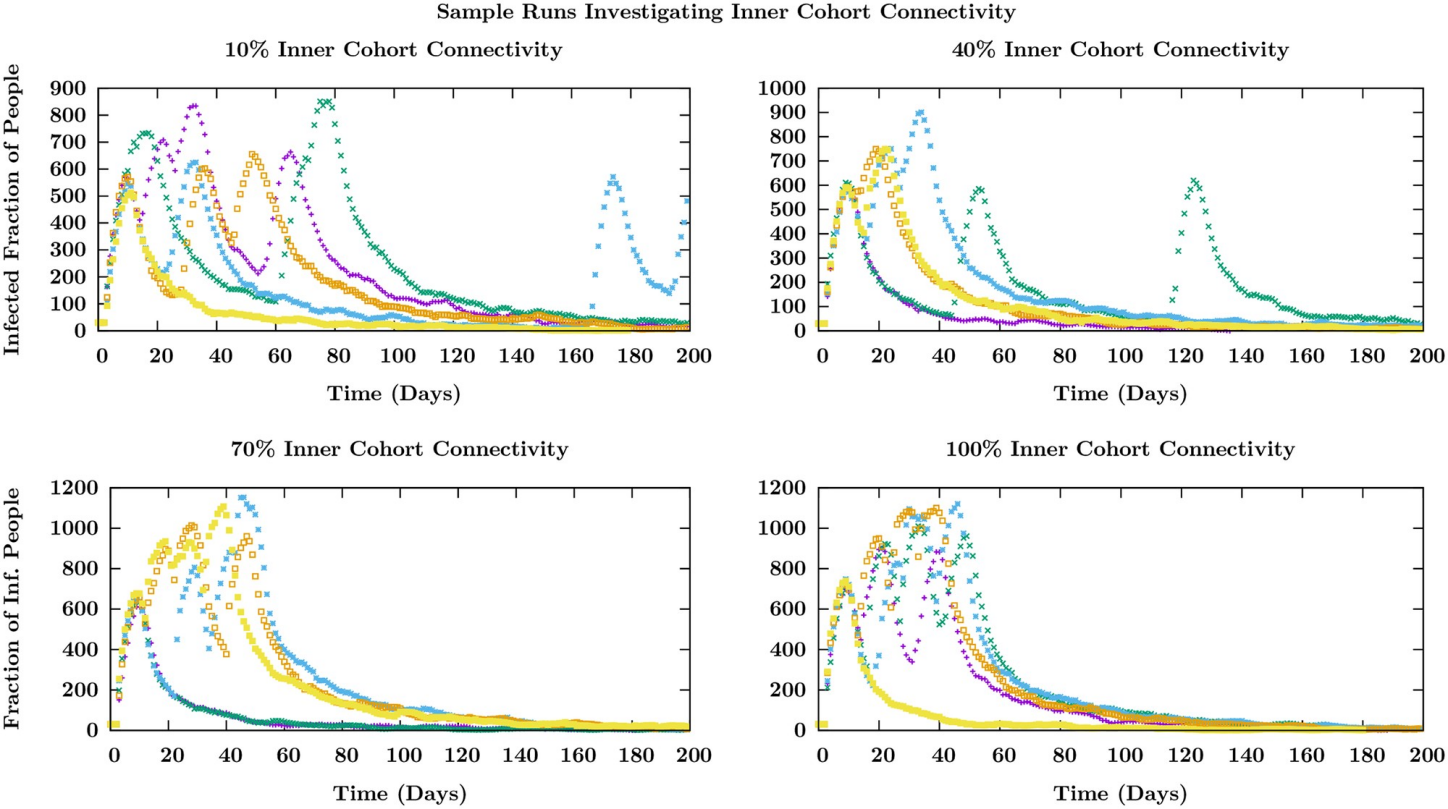
Infectious number as a function of time in several sample runs for varying degrees of inner-cohort connectivity. Different colors indicate individual samples trajectories in time which are started with the same initial condition of 30 infected individuals in the first cohort of 4 in a total population of 6000. The significant change is in the time interval between recurrence peaks as explained in the text.

We have discussed the behavior of the model when the out-degree inside the cohort is taken to be 16. When the number of intra-cohort connections is decreased substantially, the spread is slow and stochastic with the possibility of extinction. In Fig 10, we display sample runs as the number of intra-cohort connections are increased keeping the contact matrix element and other parameters unchanged. We see that at lower values of the connectivity, the peaks are separated on longer time scales and as the connectivity (the out-degree of agents within cohorts) is increased, the peaks occur closer together. The separation of the recurrences in time depends on the rate at which inter-cohort transmission occurs. This depends both on the strength of the inter-cohort connections that we have not changed, but also the speed with which the few (25) agents in one cohort who are directly connected to the next cohort become infectious. This rate depends on the speed with which the infection spreads within an individual cohort, and this time decreases as we increase the intra- (or inner) cohort connectivity. This is seen in Fig 10.

**Fig 11:**
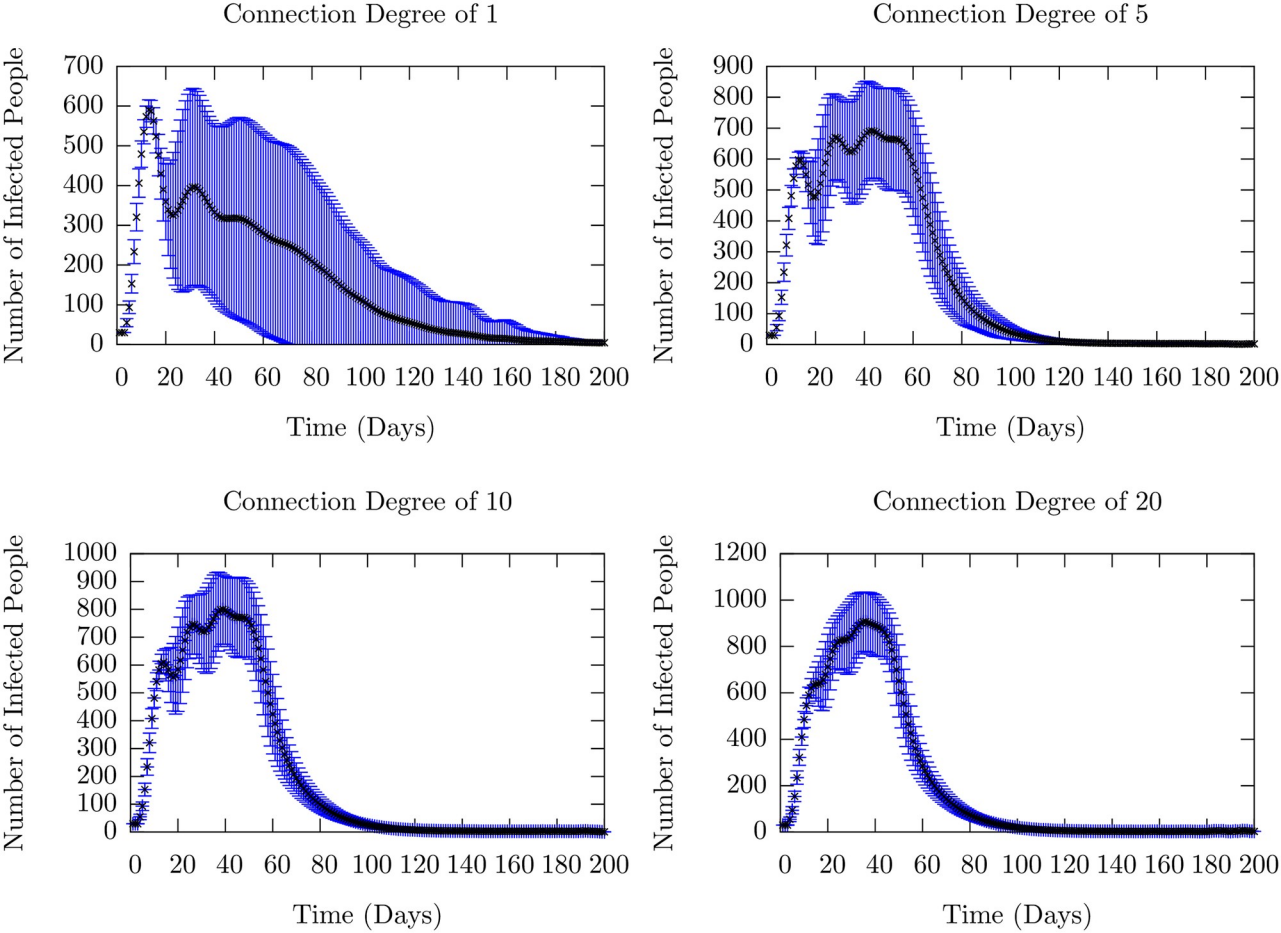
Mean number of infected (the sum of exposed and infectious) people as a function of time as the degree of inter-cohort connections is increased. The connection degree determines how many agents are connected to one of 25 randomly chosen agents in the preceding cohort. The black dots represent the ensemble mean, taken over 10,000 runs, while the blue lines represent the standard deviations. Individual runs which contributed to these averages are shown in S8 Fig. For a connection degree of 1, it is clear that a recurrence is present on average, as there are two peaks in the mean number which are clearly separated. As the number of connections drawn from an agent in the first cohort to the second are increased, we are increasing the strength of the coupling between the cohorts, as well as decreasing the average node-to-node distance between agents in different cohorts. Thus, as the connection degree is increased, we recover the single-cohort SIR-like infection curve in which there is one large period of infection.

The behavior seen in Fig 10 depends critically on the number of connections between the large cohorts, i.e. the inter-cohort degree. When each of 25 agents in the first cohort are connected to a single agent in the second (and similarly for the succeeding pairs of cohorts), it is easy to see that there is a bottleneck for the infection to spread between cohorts. However, increasing the number of agents in the succeeding cohort to whom a fraction of the agents in the preceding are connected decreases the period of time for the infection to spread between cohorts. This washes out the well-separated recurrences that correspond to spread into succeeding cohorts and one just sees the first peak. This is shown in Fig 11.

Nevertheless, since the inter-cohort transmission rate is less than the intra-cohort transmission rate, though not substantially so in these simulations, we see remnants of the recurrences within the one well-defined peak. While increasing the inter-cohort connectivity (out-degree), were we to re-scale (decrease) the connection strength correspondingly, we recover well-defined recurrences as shown in S5 Fig. The rescaling of the contact matrix element keeps the average strength of the connection between cohorts the same and similar behavior ensues.

These investigations enable us to identify quantitatively the parameter variations that affect the recurrences that are seen in the base model for which the results are displayed in Figs 3–7. In the limit that the inter-cohort and intra-cohort transmission rates become comparable, we obtain a single large infection peak as in the one cohort SEIR model. As the population is fragmented into well-separated segments, we obtain well-defined recurrence peaks corresponding to infection spreading into the successive cohorts.

### Two strains

In the 2010-2011 flu season and particularly in the 2018-2019 flu season, according to the CDC [24], two flu strains circulated in the United States. The pandemic (swine flu) H1N1 peaked early in the season, while the seasonal H3N2 surged and peaked later in the season. Motivated by these observations, we investigate a scenario within our model in which the influenza vaccine is effective against the pandemic virus that spreads first, but ineffective against the specific seasonal virus that appears later. The issue we address is the extent to which the strain that appears later, against which the population is assumed to have no induced immunity, spreads and how that depends on the fraction of the population that receives inoculation against the earlier pandemic virus.

The key model assumption is based on the following experimental result. We note that in a recent experiment [48], ex-vivo NHBE (normal human bronchial epithelial) cells were infected first with H1N1 virus, and then the H3N2 virus 509 A/Wyoming/03/2003. It was found that in bystander cells (cells that were not infected by H1N1), paracrine loops induced a strong anti-viral response (as measured by interferon-stimulated genes). Indeed, the replication of the H3N2 was decreased in comparison with single infection by H3N2. Thus, there is microscopic evidence for the influence of the immune response to the first strain on the infection by the second strain: it provides protection against it. While it is difficult to directly incorporate the results of such a microscopic model into the parameters of the macroscopic model, we assume that infection with the first virus (pandemic) provides increased immunity to the second virus (seasonal) in susceptible individuals, none of whom had received vaccinations against the seasonal flu.

This model was simulated with the following parameters. The virulence of the two strains in the model, i.e. the distributions of *V*_*i*_ of infectious individuals was kept the same in the absence of clear data to the contrary. Immunization against the pandemic strain was taken to mean that the immune strength of an inoculated individual *I*_*i*_ was uniformed randomly distributed between values 2 and 8 in contrast to [0, 6] for Individuals that are not inoculated. This implies an enhancement of the fundamental timescale of infection of inoculated individuals by at least a factor of 8.

**Fig 12:**
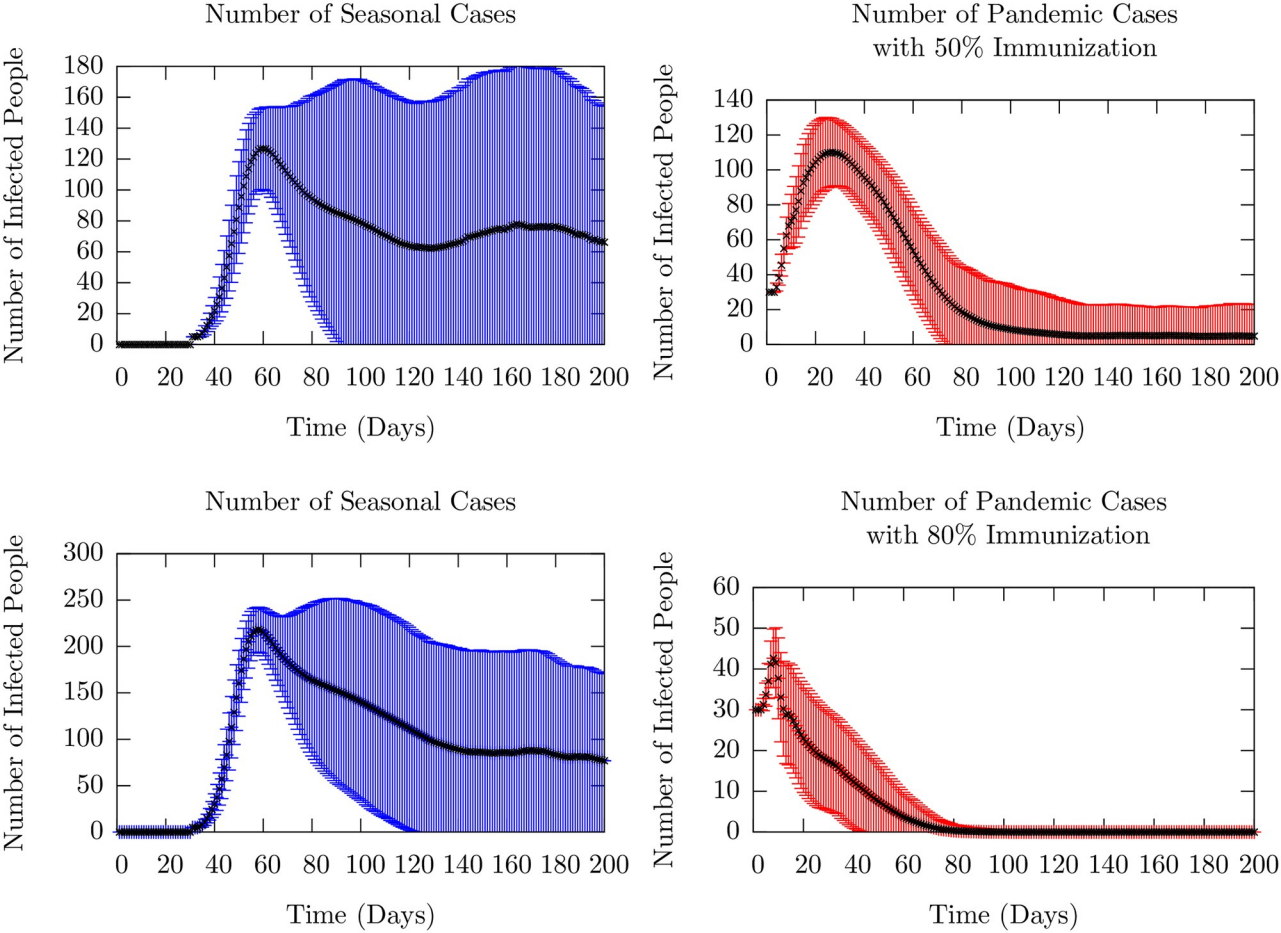
Mean infected (sum of exposed and infectious) agent number as a function of time. The black dots represent ensemble averages over 10,000 runs, and the colored bars represent the standard deviation. Individual runs which contributed to these averages are presented in S9 Fig. The left column (blue) shows the number of seasonal cases, while the right column (red) shows the number of pandemic cases. For the top row, 50% of the population was inoculated against the pandemic strain as detailed in the text, whereas in the bottom row, 80% of the population was inoculated. We see that the higher level of inoculation effectively prevents even the first peak of infection. However, this prevention is compensated by a large wave of infection in the seasonal strain; the number of peak cases is twice that of the lower inoculation rate. This feature is examined in the main text.

We investigated two cases: one with 80% inoculation rate and the other with 50% rate. We used a model with four cohorts, and a total population of *N* = 6000. The key results for the mean number of infections of the two strains as a function of time in are presented in the two panels in Fig 12. The average was taken over 10,000 runs. Individual runs for these two are shown in S9 Fig. In both cases, there is a first peak that corresponds to the initial infection due to the pandemic strain. The vaccine effectiveness over the years has ranged between 20% to 60%. We assumed that 80% or 50% of agents were inoculated, but were still susceptible to the pandemic virus, leading to the first peak. 50% inoculation is roughly equivalent to the true immunization rate in the United States as reported by the CDC [49]. We note that even though fewer agents are infected with the pandemic strain with higher inoculation, rare events can cause small recurrences at late times due to the larger pool of susceptibles.

The second strain was introduced after 30 days, roughly corresponding to the data from Columbus, Ohio from the 2018-2019 flu season [23]. We find a result that might be surprising at first sight: the number of infected cases of the seasonal strain is higher when the inoculation rate is higher for the pandemic virus. If one considers the protection afforded by being infected by the pandemic virus and the model population structure (specifically, the contact matrix that allows only few, weaker links between the cohorts) the result can be understood. If the small number of agents that provide connectivity between cohorts are infected by the first virus (more likely with the lower inoculation rate), then they cannot be infected by the seasonal virus. This reduces the probability of transmission to the succeeding cohorts and thus limits the number of seasonal infections. Once again, we chose parameters to make peaks clearly visible in order to study these effects. We note also the increase in stochasticity of the occurrence of the seasonal infection seen in the curves in the lower right panel of S9 Fig; this is a result of the rare events that control the inter-cohort spread due to the weak connections between cohorts. We emphasize, however, that we have pointed out a possible mechanism that can yield an increase the infection number for the second strain when vaccination rate for the first strain is increased.

**Fig 13:**
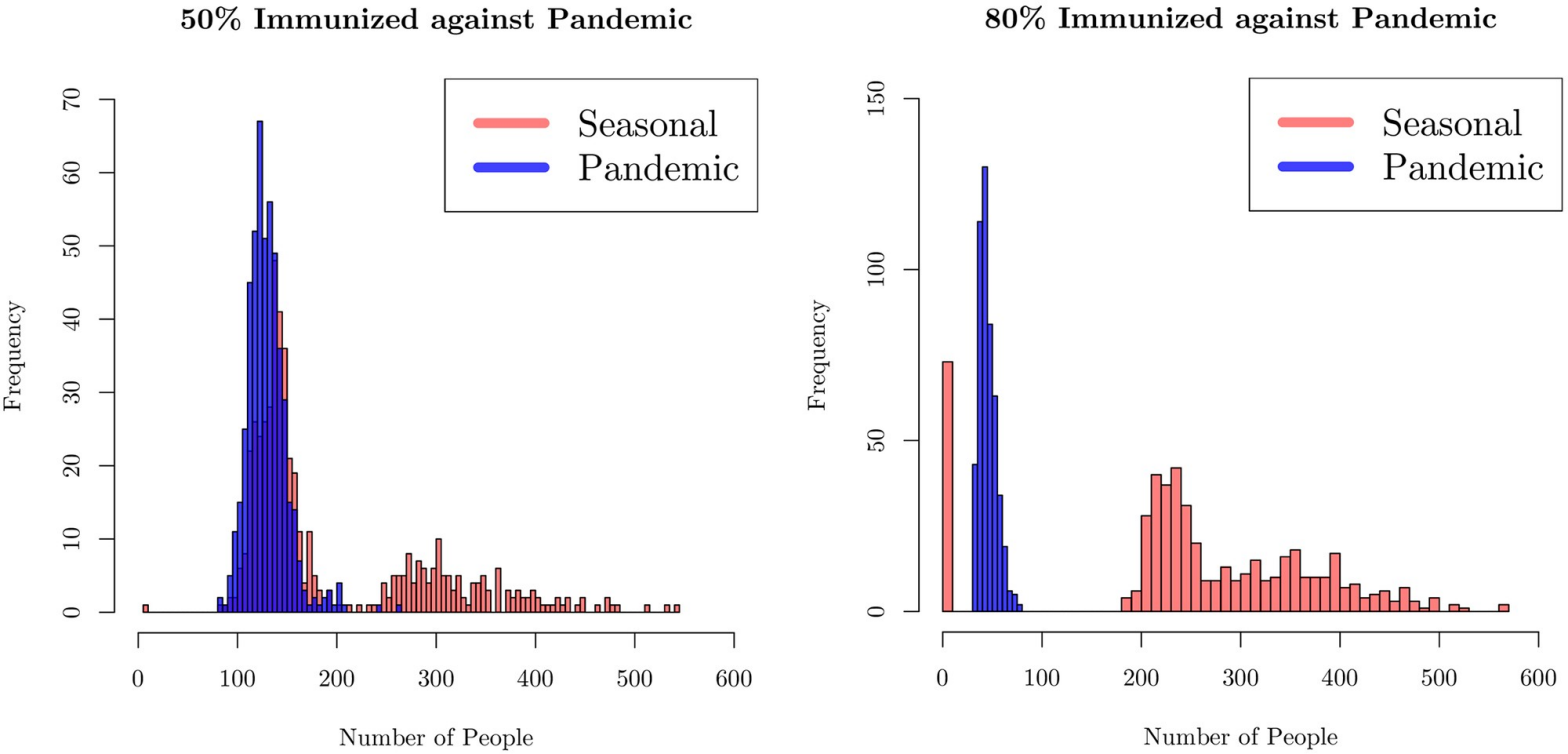
Histograms of the maximum number (defined as the highest number at a given time over the entire season) of infected people with either the seasonal or pandemic strain of influenza in a season. In the left figure, half of the population has been inoculated against the pandemic strain, whereas in the right figure, 80% of the population has been inoculated. We see a natural reduction in the number of pandemic cases, but also a rightward shift in the number of seasonal cases, indicating that a higher level of immunization to the pandemic strain increases the susceptibility of the population at large to the seasonal strain.

In Fig 13, we display histograms of the maximum number of people infected with each of the strains in each sample. The left panel shows the probability of a given maximum infection level for the case with no immunization against the seasonal strain and 50% coverage against the pandemic. The figure in the right panel is for the case of 80% coverage of immunization against the pandemic that occurs earlier. The peak numbers of those infected with the pandemic strain (the blue histogram) declines with increase in the immunization level. While larger number of agents become infected with the seasonal strain compared to the pandemic strain infections in both cases, the contrast is clear. The maximum number of people infected by the seasonal strain (orange-red histogram) has significantly larger weight at larger numbers. Since a larger fraction of the population is protected from pandemic infection for higher immunization, they are more susceptible to the seasonal strain, leading to larger epidemic spreads.

## Discussion

In this paper, we have presented results for the time course of the number of infected individuals from a numerical simulation study of a simple agent-based model of influenza spread. We have investigated the effect of the topology of connections between members of a population and the strength of the immune profile of individuals due to immunization. We identified quantitatively the parameter variations that affect the recurrences and showed that as the population is fragmented into cohorts which are more and more weakly connected, we obtain increasingly better-defined recurrence peaks corresponding to infection spreading into the successive cohorts.

We examined the stochastic nature of recurrence peaks in detail: rare events, arising from weak transmissibilty between cohorts of populations, leading to opportunistic spread of infection from one cohort to another give rise to recurrences that can occur randomly. Within a cohort whose members are well-immunized, although the infectious number is limited, the times at which the peak occurs can be broadly distributed. The study of a two-strain model revealed that immunity induced by inoculation against one strain of infection can lead to increased occurrence of a second later infection. The simplicity of the model allowed us to conduct a detailed study of its behavior, leaving open for future work the consequences of including specific effects such as time-varying environmental influences.

## Supporting information

S1 Fig *I*_1_ + *I*_2_ in the ODE SIR model with time delay. There is a delay inserted between the time of infection and the transition of the susceptible number to infected. The model is solved with the initial conditions of 0.5% of the total population in the first cohort infected, with the remaining population being susceptible. We see the same oscillatory behavior as seen for the case of the SEIR model shown in Fig 2.
(TIF)

S2 Fig Histogram of the number of peaks found in the infection time course over 1000 runs of the case of weak population immune strength. The parameters are those used in Fig 3. Peaks were found using FindPeaks, a Mathematica utility, after being smoothed by Wiener filtering. More than three-quarters of the samples have more than one peak in this case in contrast to the strongly immune case shown in S3 Fig.
(TIF)

S3 Fig Histogram of the number of peaks found in the infection time course over 1000 runs of the case of strong population immune strength. The parameters are those used in Fig 4. Peaks were found using FindPeaks, a Mathematica utility, after being smoothed by Wiener filtering. More than half the samples in this strongly immune case have only one peak in contrast to the weakly immune case in S2 Fig.
(TIF)

S4 Fig Sample runs of the weak immune population immune strength case with a population size of 20,000. We see clear, stable recurrences, in accordance with the large *N* behavior shown S7 Fig. All other parameters are those given in Table 1.
(TIF)

S5 Fig Sample runs of the model investigating the degree of inter-cohort connectivity with connection strength scaled with inverse connection size. We see, in contrast to the situation where the connection strength is not scaled (see Fig 11), we retain stable recurrences throughout the increase in inter-cohort connectivity.
(TIF)

S6 Fig Histogram of incidence of first infection in the second cohort for a strong immune response. As discussed in the text, the low probability of the spread leads to very few samples (5%) showing the second cohort getting infected and the distribution is determined by the length of the season. The corresponding figure for weak immune response is in the left panel of Fig 6.
(TIF)

S7 Fig Individual samples contributing to the mean time course of infected number in Fig 9. We see that as *N* increases, the mean length of the run increases. At *N* = 1000, samples undergo stochastic extinction. At *N* = 2000, the extinction occurs after a long tail with some runs exhibiting a small recurrence. At *N* = 4000 and above, the recurrences are stable.
(TIF)

S8 Fig Analysis of the model as the inter-cohort connection degree increases. The behavior of the mean number is shown in Fig 11. Different colors correspond to individual runs which began with the same initial conditions of 30 people infected in a population of 6000. The trend we point out is that the recurrences which are well-defined and separated for low connection degree become smoothed into one larger infection peak as the degree is progressively increased. Since this increase leads to a much more well-connected population both inside and across cohorts, the model tends to the results for the single-cohort ODE SEIR model in this limit.
(TIF)

S9 Fig Sample runs of the infected number for the pandemic strain (left) and seasonal strain (right) for the two cases with low 50% and high 80% inoculation rates in the top and bottom rows respectively. The mean infected number dynamics for the corresponding parameters is shown in Fig 12. As expected, with lower levels of immunization, the number of people infected with pandemic flu increases. However, we observe the suppression of infection recurrences in the seasonal strain for lower levels of vaccination against the pandemic strain (b). In contrast, in the lower right panel for the higher rate of inoculation for the pandemic strain, the seasonal strain can spread more widely throughout the cohorts. This can be understood as a result of the immunity to the second, seasonal infection conferred by being infected earlier by first pandemic strain as explained in the text. The structure of the contact matrix is given in the model section, and virulence parameters are given in Table 1.
(TIF)

## References

[1] Brachman PS. Infectious diseases—past, present, and future. International Journal of Epidemiology. 2003;32(5):684–686. 10.1093/ije/dyg282 14559728

[2] Fonkwo PN. Pricing infectious disease. EMBO reports. 2008;9(S1):S13–S17. 10.1038/embor.2008.110 18578017 PMC3327542

[3] Iuliano AD, Roguski KM, Chang HH, Muscatello DJ, Palekar R, Tempia S, et al Estimates of global seasonal influenza-associated respiratory mortality: a modelling study. The Lancet. 2018;391(10127):1285–1300. 10.1016/S0140-6736(17)33293-2 PMC5935243 29248255

[4] Tokars JI, Olsen SJ, Reed C. Seasonal Incidence of Symptomatic Influenza in the United States. Clinical Infectious Diseases. 2017;66(10):1511–1518. 10.1093/cid/cix1060 PMC5934309 29206909

[5] Caini S, Kroneman M, Wiegers T, El Guerche-Séblain C, Paget J. Clinical characteristics and severity of influenza infections by virus type, subtype, and lineage: A systematic literature review. Influenza and Other Respiratory Viruses. 2018;12(6):780–792. 10.1111/irv.12575 29858537 PMC6185883

[6] Kermack WO, McKendrick AG, Walker GT. A contribution to the mathematical theory of epidemics. Proceedings of the Royal Society of London Series A, Containing Papers of a Mathematical and Physical Character. 1927;115(772):700–721.

[7] Keeling MJ, Rohani P. Modeling infectious diseases in humans and animals. Princeton University Press; 2008.

[8] Kilbourne ED. Influenza pandemics of the 20th century. Emerging infectious diseases. 2006;12(1):9–14. 10.3201/eid1201.051254 16494710 PMC3291411

[9] Venkatramanan S, Lewis B, Chen J, Higdon D, Vullikanti A, Marathe M. Using data-driven agent-based models for forecasting emerging infectious diseases. Epidemics. 2018;22:43—49. 10.1016/j.epidem.2017.02.010 28256420 PMC5568513

[10] Chao DL, Halloran ME, Obenchain VJ, Longini IM Jr. FluTE, a Publicly Available Stochastic Influenza Epidemic Simulation Model. PLOS Computational Biology. 2010;6(1):1–8. 10.1371/journal.pcbi.1000656 PMC2813259 20126529

[11] Bartlett MS. Stochastic population models in ecology and epidemiology. Methuen and Co.; 1970.

[12] Bailey NTJ. The mathematical theory of epidemics. Griffin; 1977.

[13] Rvachev LA. Modelling experiment of a large-scale epidemic by means of a computer. Dokl Akad Nauk SSSR. 1968;180(2):294–296.

[14] Watts DJ, Muhamad R, Medina DC, Dodds PS. Multiscale, resurgent epidemics in a hierarchical metapopulation model. Proceedings of the National Academy of Sciences. 2005;102(32):11157–11162. 10.1073/pnas.0501226102 PMC1183543 16055564

[15] Gómez S, Fernández A, Meloni S, Arenas A. Impact of origin-destination information in epidemic spreading. Scientific Reports. 2019;9(1):2315 10.1038/s41598-019-38722-4 30783178 PMC6381217

[16] Tanaka G, Urabe C, Aihara K. Random and Targeted Interventions for Epidemic Control in Metapopulation Models. Scientific Reports. 2014;4(1):5522 10.1038/srep05522 25026972 PMC4099978

[17] Guo D, Li KC, Peters TR, Snively BM, Poehling KA, Zhou X. Multi-scale modeling for the transmission of influenza and the evaluation of interventions toward it. Scientific Reports. 2015;5:8980 EP –. 10.1038/srep08980 25757402 PMC4355742

[18] Fox SJ, Miller JC, Meyers LA. Seasonality in risk of pandemic influenza emergence. PLOS Computational Biology. 2017;13(10):1–23. 10.1371/journal.pcbi.1005749 PMC5654262 29049288

[19] Roche B, Drake JM, Rohani P. An Agent-Based Model to study the epidemiological and evolutionary dynamics of Influenza viruses. BMC Bioinformatics. 2011;12(1):87 10.1186/1471-2105-12-87 21450071 PMC3078862

[20] Longini IM, Nizam A, Xu S, Ungchusak K, Hanshaoworakul W, Cummings DAT, et al Containing Pandemic Influenza at the Source. Science. 2005;309(5737):1083–1087. 10.1126/science.1115717 16079251

[21] for Disease Control C, Prevention. Weekly U.S. Influenza Surveillance Report; 2019. Available from: https://www.cdc.gov/flu/weekly/index.htm.

[22] of Health OD. Ohio Flu Season Data; 2019. Available from: https://odh.ohio.gov/wps/portal/gov/odh/know-our-programs/seasonal-influenza/ohio-flu-activity/ohio-flu-activity.

[23] of Columbus C. Columbus Flu Reports. Influenza Surveillance. 2019. Available from: https://www.columbus.gov/Weekly_Flu_Update/.

[24] for Disease Control C, Prevention. Influenza Information. Centers for Disease Control and Prevention. 2018. Available from: https://www.cdc.gov/flu/about/disease/spread.htm.

[25] Bartlett MS. Deterministic and Stochastic Models for Recurrent Epidemics. In: Proceedings of the Third Berkeley Symposium on Mathematical Statistics and Probability, Volume 4: Contributions to Biology and Problems of Health. Berkeley, Calif.: University of California Press; 1956. p. 81–109. Available from: https://projecteuclid.org/euclid.bsmsp/1200502549.

[26] Soper HE. The Interpretation of Periodicity in Disease Prevalence. Journal of the Royal Statistical Society. 1929;92(1):34–73. 10.2307/2341437

[27] Bartlett MS. Measles Periodicity and Community Size. Journal of the Royal Statistical Society: Series A (General). 1957;120(1):48–60. 10.2307/2342553

[28] Lessler J, Reich NG, Brookmeyer R, Perl TM, Nelson KE, Cummings DA. Incubation periods of acute respiratory viral infections: a systematic review. The Lancet Infectious Diseases. 2009;9(5):291–300. 10.1016/S1473-3099(09)70069-6 19393959 PMC4327893

[29] Sanjuán R, Nebot MR, Chirico N, Mansky LM, Belshaw R. Viral Mutation Rates. Journal of Virology. 2010;84(19):9733–9748. 10.1128/JVI.00694-10 20660197 PMC2937809

[30] Lukens S, DePasse J, Rosenfeld R, Ghedin E, Mochan E, Brown ST, et al A large-scale immuno-epidemiological simulation of influenza A epidemics. BMC Public Health. 2014;14(1):1019 10.1186/1471-2458-14-1019 25266818 PMC4194421

[31] Woodhouse DE, Rothenberg RB, Potterat JJ, Darrow WW, Muth SQ, Klovdahl AS, et al Mapping a social network of heterosexuals at high risk for HIV infection. AIDS. 1994;8(9):1331–1336. 10.1097/00002030-199409000-00018 7802989

[32] WYLIE JL, JOLLY A. Patterns of Chlamydia and Gonorrhea Infection in Sexual Networks in Manitoba, Canada. Sexually Transmitted Diseases. 2001;28(1). 10.1097/00007435-200101000-00005 11196040

[33] Rothenberg RB, Potterat JJ, Woodhouse DE, Muth SQ, Darrow WW, Klovdahl AS. Social network dynamics and HIV transmission. AIDS. 1998;12(12). 10.1097/00002030-199812000-00016 9727575

[34] Riley S, Fraser C, Donnelly CA, Ghani AC, Abu-Raddad LJ, Hedley AJ, et al Transmission Dynamics of the Etiological Agent of SARS in Hong Kong: Impact of Public Health Interventions. Science. 2003;300(5627):1961–1966. 10.1126/science.1086478 12766206

[35] Uyeki TM, Bernstein HH, Bradley JS, Englund JA, File J Thomas M, Fry AM, et al Clinical Practice Guidelines by the Infectious Diseases Society of America: 2018 Update on Diagnosis, Treatment, Chemoprophylaxis, and Institutional Outbreak Management of Seasonal Influenzaa. Clinical Infectious Diseases. 2018;68(6):e1–e47. 10.1093/cid/ciy866 PMC6653685 30566567

[36] Pulendran B, Li S, Nakaya HI. Systems Vaccinology. Immunity. 2010;33(4):516—529. 10.1016/j.immuni.2010.10.006 21029962 PMC3001343

[37] Pulendran B. Systems vaccinology: Probing humanity’s diverse immune systems with vaccines. Proceedings of the National Academy of Sciences. 2014;111(34):12300–12306. 10.1073/pnas.1400476111 PMC4151766 25136102

[38] Wolfram Research, Inc. Mathematica, Version 12.0 Available from: https://www.wolfram.com/mathematica Champaign, IL, 2019.

[39] Press WH, Teukolsky SA, Vetterling WT, Flannery BP. Numerical recipes in C. Cambridge Univ. Pr.; 1992.

[40] Research W. FindPeaks Documentation FindPeaks-Wolfram Language Documentation. 2019;.

[41] Murillo LN, Murillo MS, Perelson AS. Towards multiscale modeling of influenza infection. Journal of Theoretical Biology. 2013;332:267—290. 10.1016/j.jtbi.2013.03.024 23608630 PMC3758892

[42] Smith H. An Introduction to Delay Differential Equations with Applications to the Life Sciences. Springer; 2010.

[43] Carrat F, Vergu E, Ferguson NM, Lemaitre M, Cauchemez S, Leach S, et al Time Lines of Infection and Disease in Human Influenza: A Review of Volunteer Challenge Studies. American Journal of Epidemiology. 2008;167(7):775–785. 10.1093/aje/kwm375 18230677

[44] Bernstein SL, Aronsky D, Duseja R, Epstein S, Handel D, Hwang U, et al The Effect of Emergency Department Crowding on Clinically Oriented Outcomes. Academic Emergency Medicine. 2009;16(1):1–10. 10.1111/j.1553-2712.2008.00295.x 19007346

[45] Segel LA, Slemrod M. The Quasi-Steady-State Assumption: A Case Study in Perturbation. SIAM Review. 1989;31(3):446–477. 10.1137/1031091

[46] Lande R. Demographic Stochasticity and Allee Effect on a Scale with Isotropic Noise. Oikos. 1998;83(2):353–358. 10.2307/3546849

[47] Caravagna G, d’Onofrio A, Milazzo P, Barbuti R. Tumour suppression by immune system through stochastic oscillations. Journal of Theoretical Biology. 2010;265(3):336—345. 10.1016/j.jtbi.2010.05.013 20580640

[48] Ramos I, Smith G, Ruf-zamojski F, Martinez-Romero C, Fribourg M, Carbajal EA, et al Innate immune response to influenza virus at single-cell resolution in human epithelial cells revealed paracrine induction of interferon lambda 1. Journal of Virology. 2019;. 10.1128/JVI.00559-19 PMC6798124 31375585

[49] for Disease Control C. CDC Sortable Stats. CDC Published Data. 2019. Available from: https://sortablestats.cdc.gov/#/.

