# Supplementary figures and images for: Stochastic modeling of influenza spread dynamics with recurrences
Source: PLoS One. 2020 Apr 21;15(4):e0231521. doi: 10.1371/journal.pone.0231521 (PMC7173783; doi:10.1371/journal.pone.0231521)

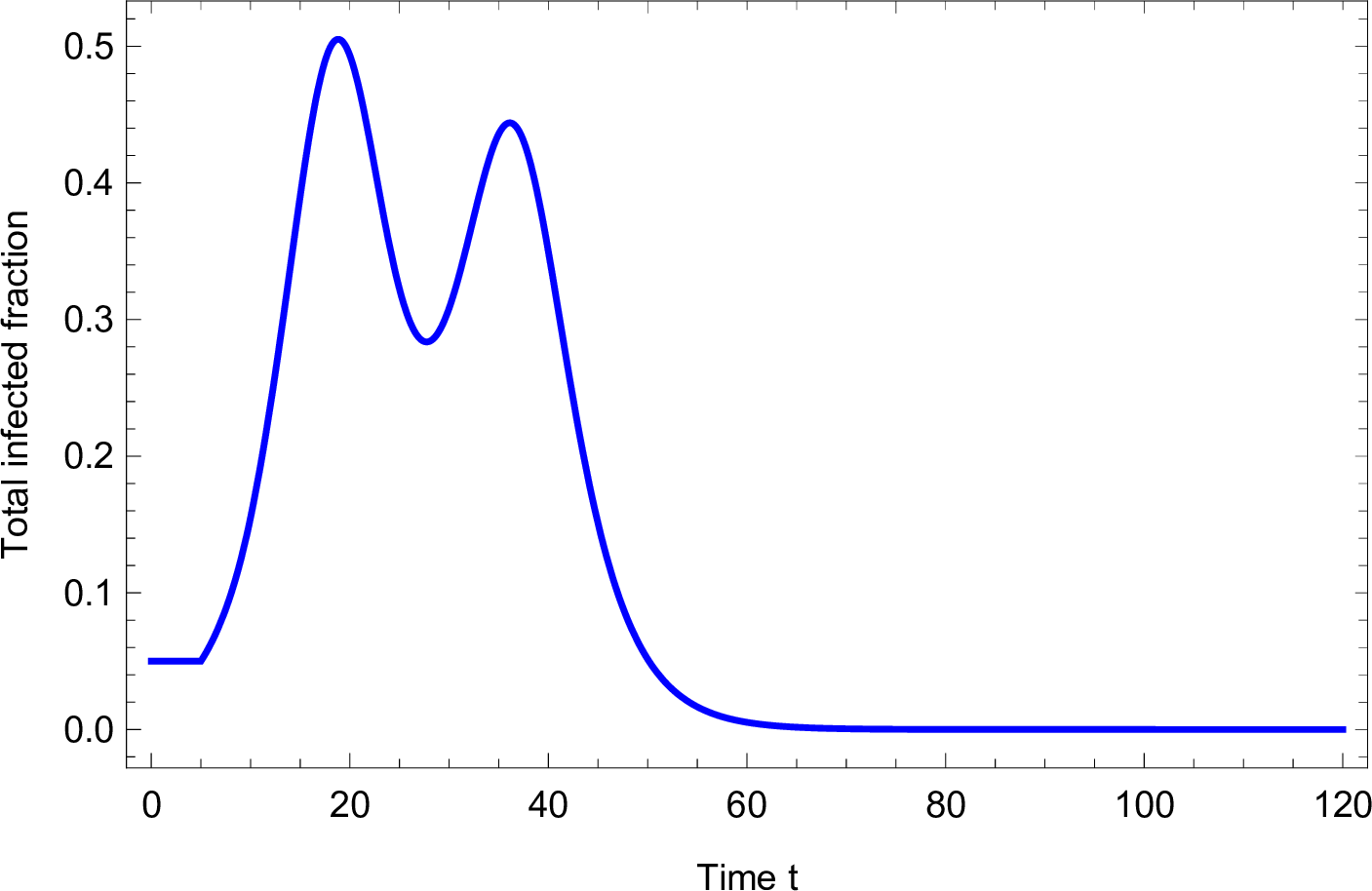

Supplement: S1 Fig — There is a delay inserted between the time of infection and the transition of the susceptible number to infected. The model is solved with the initial conditions of 0.5% of the total population in the first cohort infected, with the remaining population being susceptible. We see the same oscillatory behavior as seen for the case of the SEIR model shown in Fig 2. (TIF) [file pone.0231521.s001.tif]

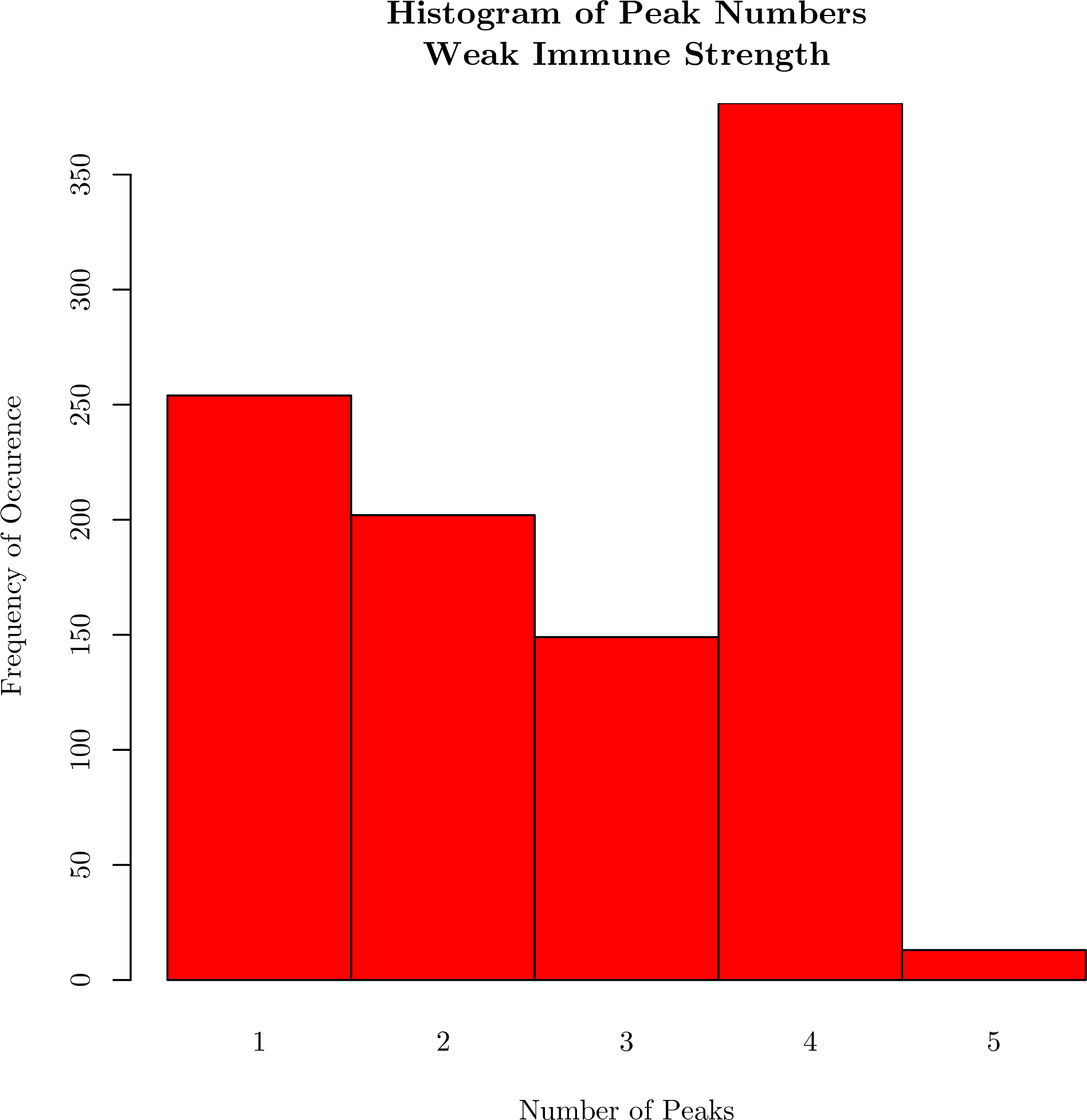

Supplement: S2 Fig — The parameters are those used in Fig 3. Peaks were found using FindPeaks, a Mathematica utility, after being smoothed by Wiener filtering. More than three-quarters of the samples have more than one peak in this case in contrast to the strongly immune case shown in S3 Fig. (TIF) [file pone.0231521.s002.tif]

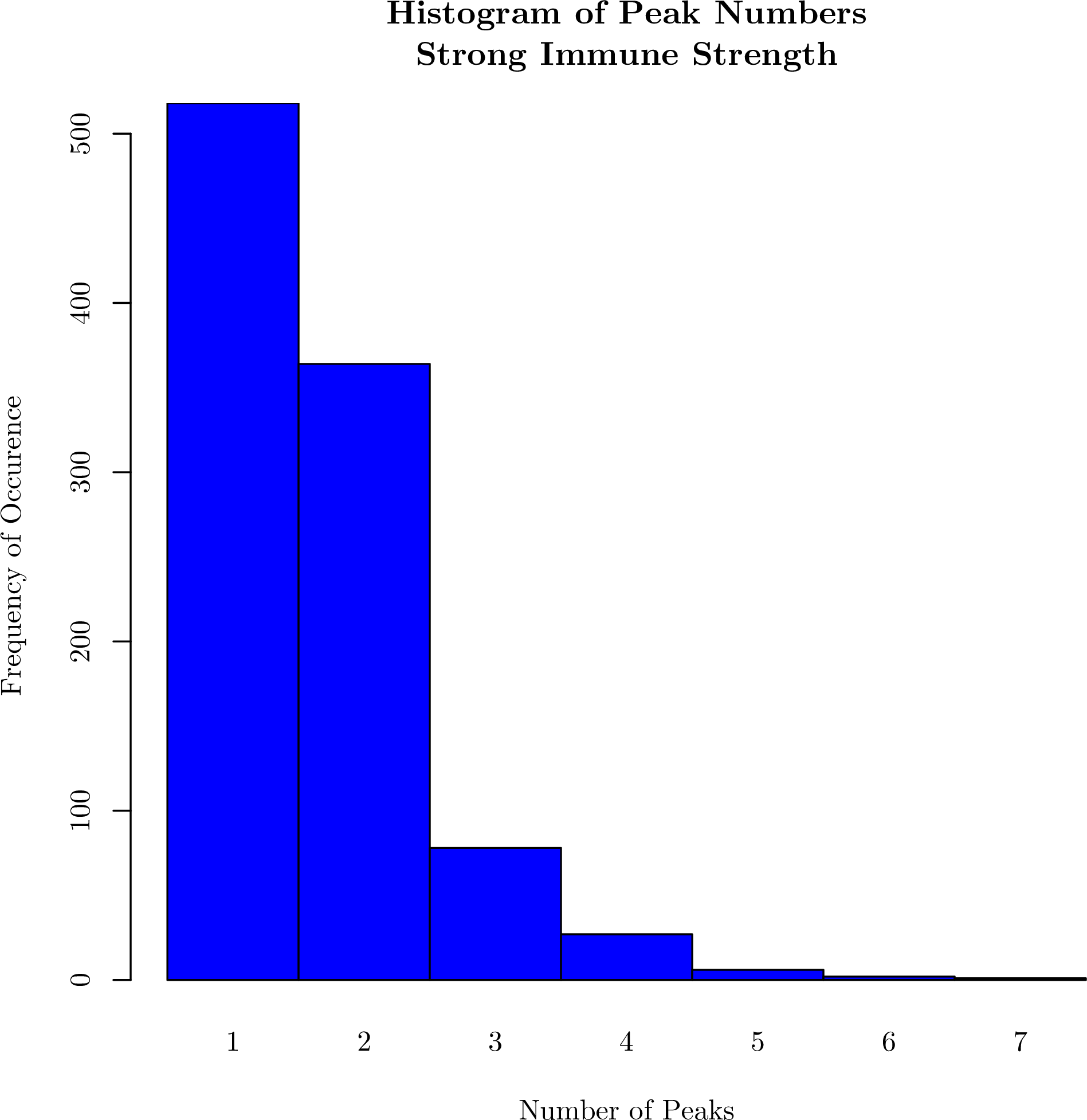

Supplement: S3 Fig — The parameters are those used in Fig 4. Peaks were found using FindPeaks, a Mathematica utility, after being smoothed by Wiener filtering. More than half the samples in this strongly immune case have only one peak in contrast to the weakly immune case in S2 Fig. (TIF) [file pone.0231521.s003.tif]

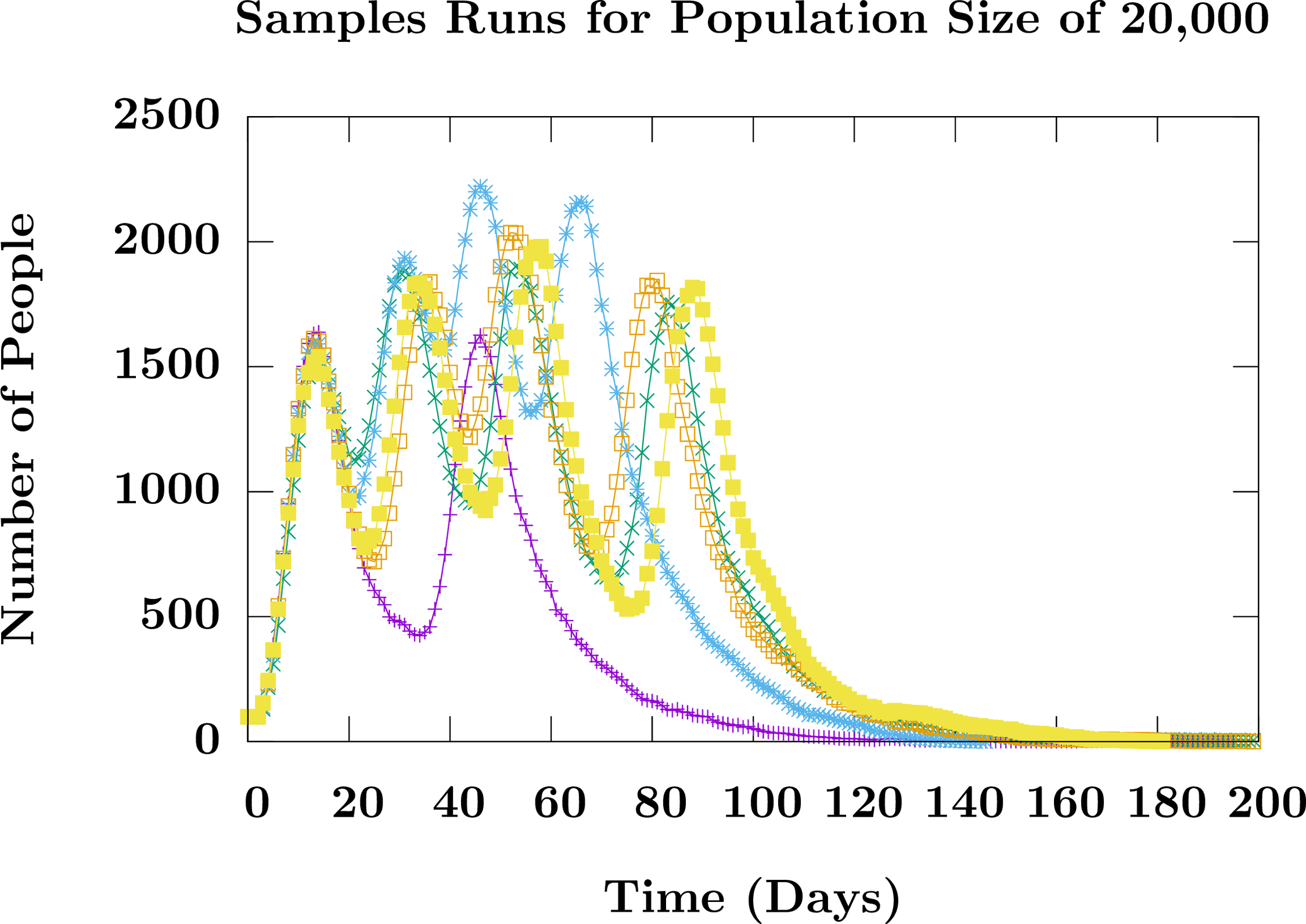

Supplement: S4 Fig — We see clear, stable recurrences, in accordance with the large N behavior shown S7 Fig. All other parameters are those given in Table 1. (TIF) [file pone.0231521.s004.tif]

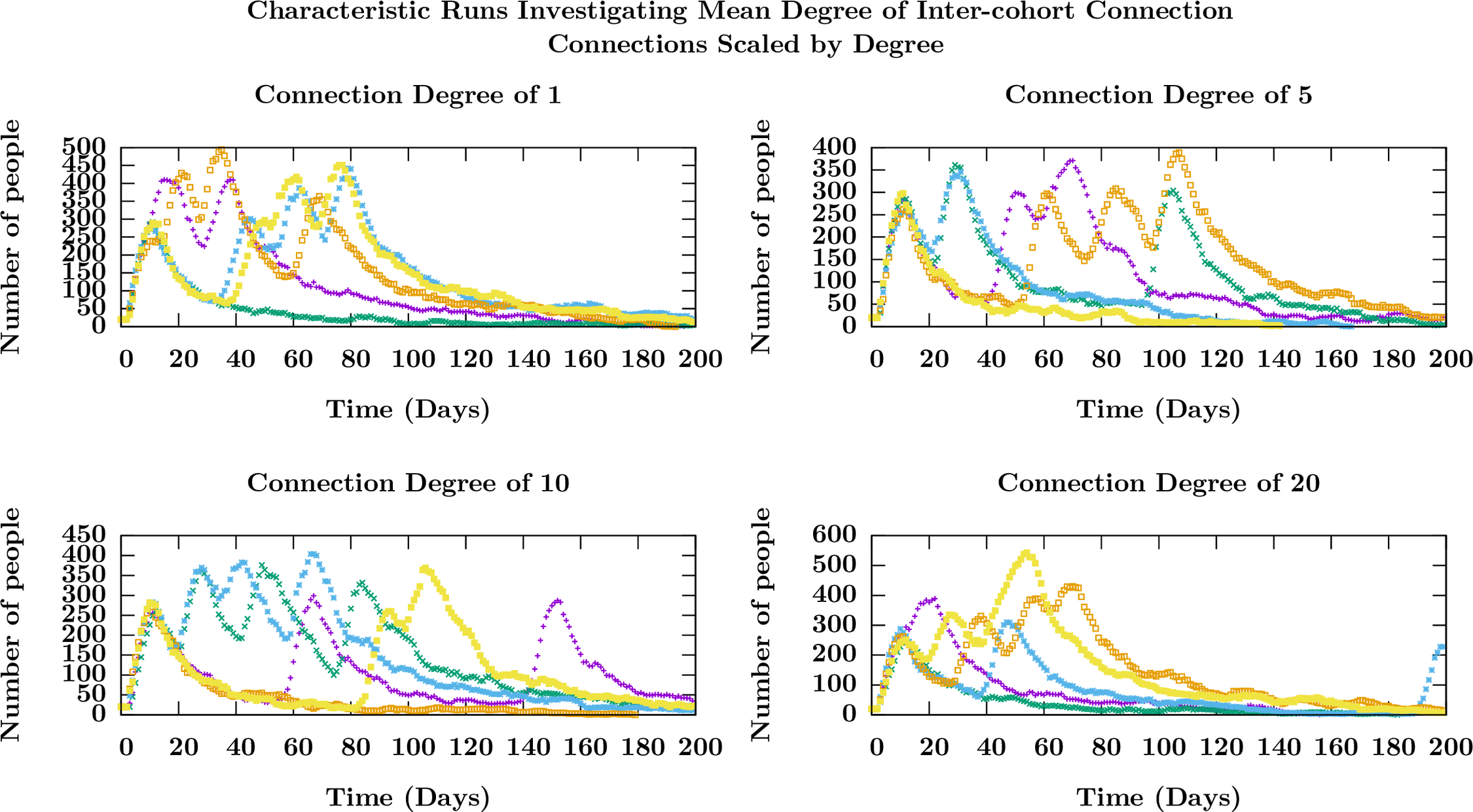

Supplement: S5 Fig — We see, in contrast to the situation where the connection strength is not scaled (see Fig 11), we retain stable recurrences throughout the increase in inter-cohort connectivity. (TIF) [file pone.0231521.s005.tif]

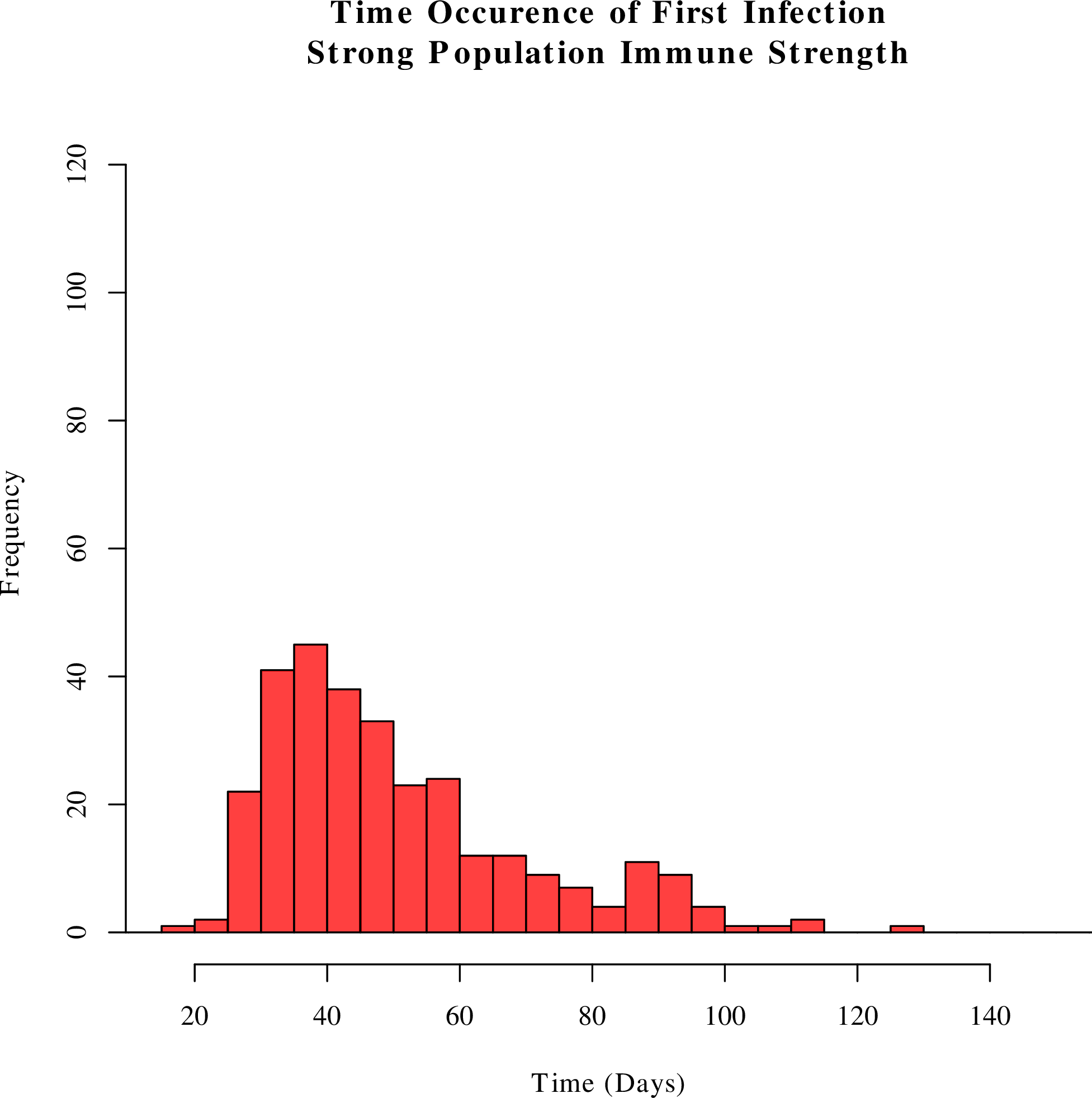

Supplement: S6 Fig — As discussed in the text, the low probability of the spread leads to very few samples (5%) showing the second cohort getting infected and the distribution is determined by the length of the season. The corresponding figure for weak immune response is in the left panel of Fig 6. (TIF) [file pone.0231521.s006.tif]

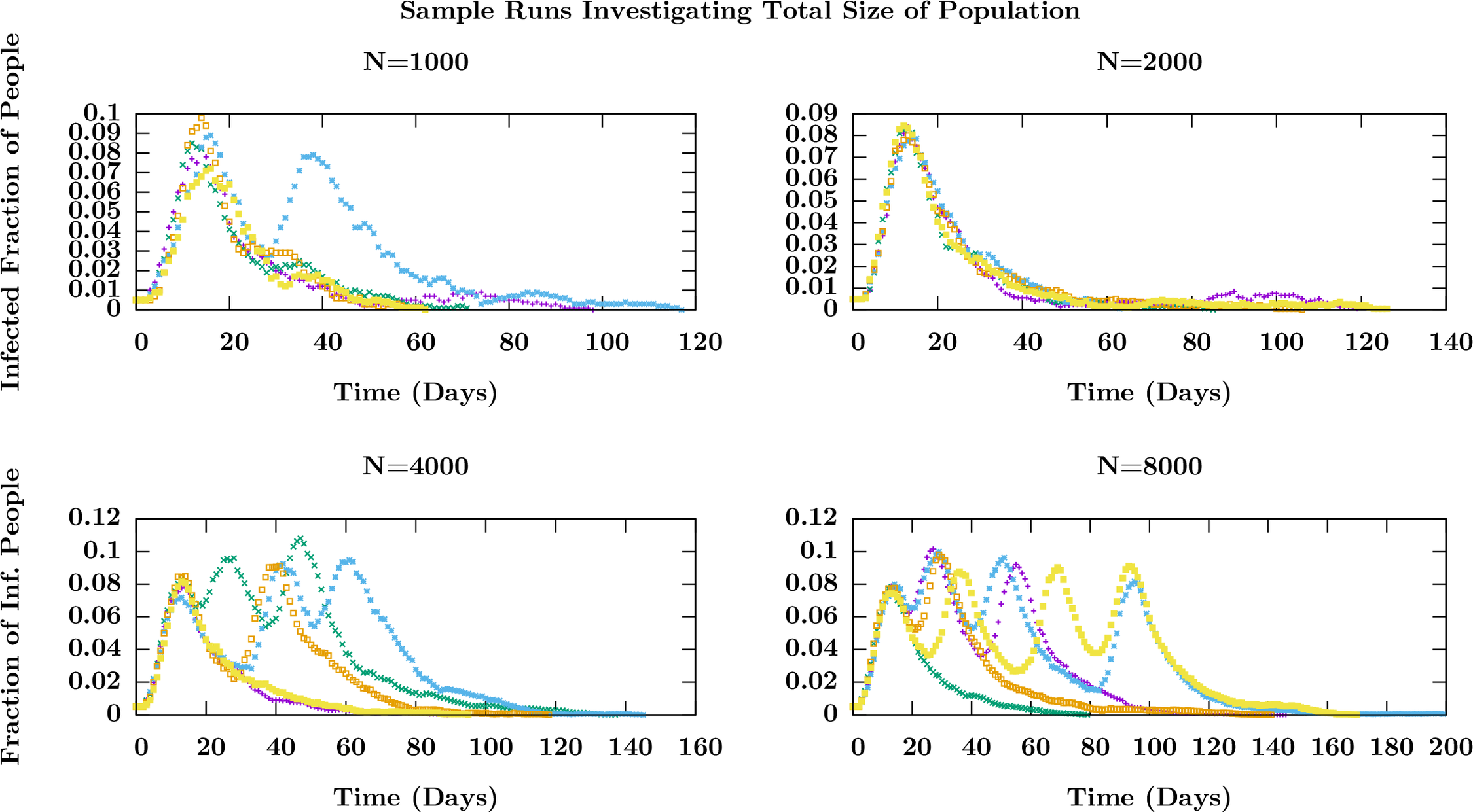

Supplement: S7 Fig — We see that as N increases, the mean length of the run increases. At N = 1000, samples undergo stochastic extinction. At N = 2000, the extinction occurs after a long tail with some runs exhibiting a small recurrence. At N = 4000 and above, the recurrences are stable. (TIF) [file pone.0231521.s007.tif]

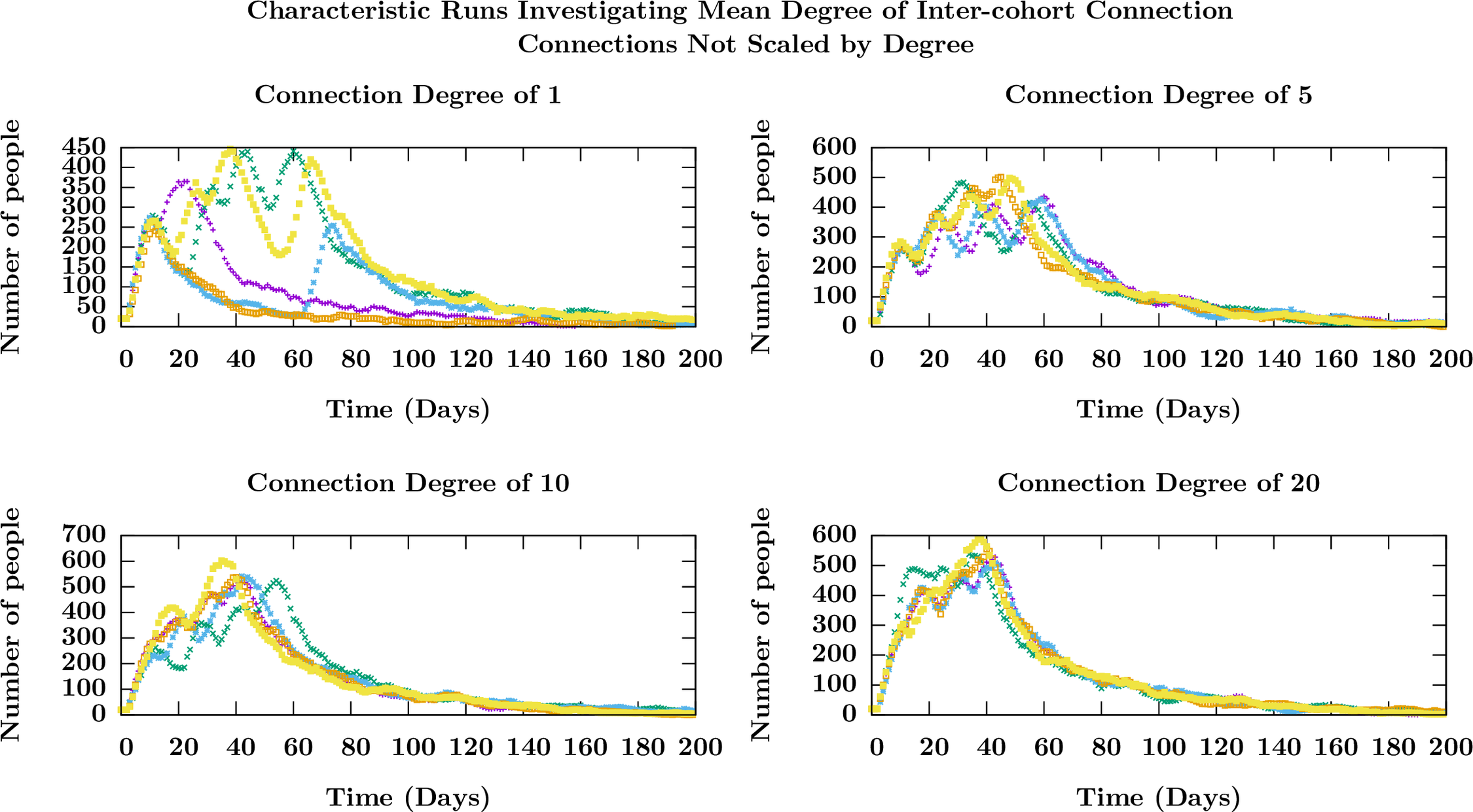

Supplement: S8 Fig — The behavior of the mean number is shown in Fig 11. Different colors correspond to individual runs which began with the same initial conditions of 30 people infected in a population of 6000. The trend we point out is that the recurrences which are well-defined and separated for low connection degree become smoothed into one larger infection peak as the degree is progressively increased. Since this increase leads to a much more well-connected population both inside and across cohorts, the model tends to the results for the single-cohort ODE SEIR model in this limit. (TIF) [file pone.0231521.s008.tif]

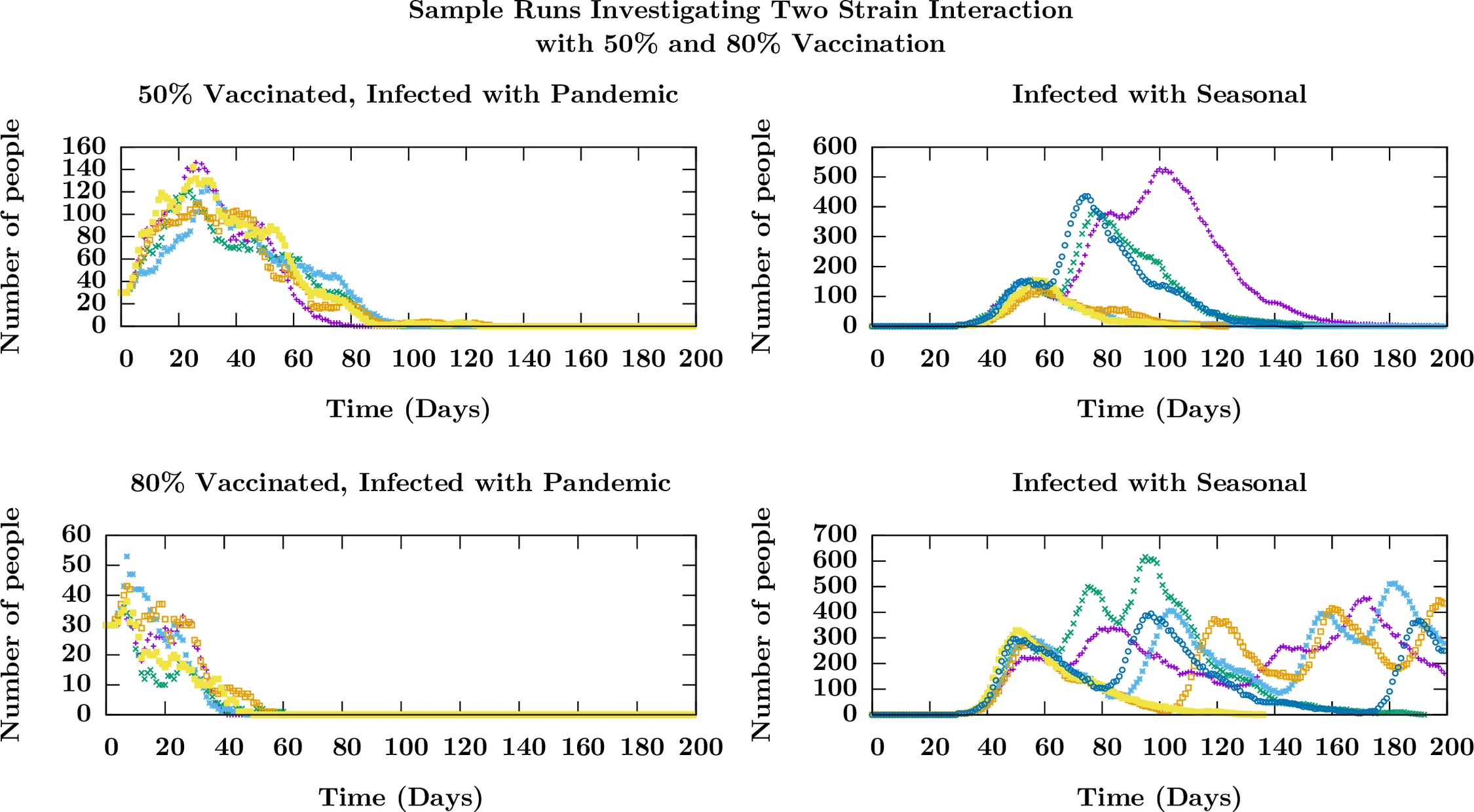

Supplement: S9 Fig — The mean infected number dynamics for the corresponding parameters is shown in Fig 12. As expected, with lower levels of immunization, the number of people infected with pandemic flu increases. However, we observe the suppression of infection recurrences in the seasonal strain for lower levels of vaccination against the pandemic strain (b). In contrast, in the lower right panel for the higher rate of inoculation for the pandemic strain, the seasonal strain can spread more widely throughout the cohorts. This can be understood as a result of the immunity to the second, seasonal infection conferred by being infected earlier by first pandemic strain as explained in the text. The structure of the contact matrix is given in the model section, and virulence parameters are given in Table 1. (TIF) [file pone.0231521.s009.tif]
